# On Low-Rank Multiplicity-Free Complex Fusion Categories

**DOI:** 10.1007/s00220-026-05705-2

**Published:** 2026-09-27

**Authors:** Gert Vercleyen, Joost Slingerland

**Affiliations:** 1 Department of Mathematics, Purdue University, 150 N University St, West Lafayette, 47907 IN USA; 2 Department of Physics, Maynooth University, Maynooth, Kildare, W23 HW31 Ireland

## Abstract

This paper explains the methods and algorithms we used to obtain explicit *F*-symbols, *R*-symbols, and pivotal coefficients of what we believe are all multiplicity-free pivotal complex fusion categories up to rank 7. Particular attention is given to how the individual steps for solving the consistency equations are done with Anyonica, a software package we developed for working with fusion categories. Gauge and automorphism equivalence are reviewed and a method to remove equivalent categories via a gauge-split basis is given. A census of all multiplicity-free fusion categories up to rank 7, along with all compatible braided and pivotal structures, is presented, and comments on their general properties are given.

## Introduction

Fusion categories serve as unifying threads connecting diverse branches of mathematics and theoretical physics [1]. They have proven useful in bridging the gap between many research areas, such as topological quantum computation [2–6], knot theory [7–10], representation theory of weak Hopf algebras and quantum groups [7, 11], low-dimensional topology and topological field theory [11–15], subfactor theory and planar algebras [16–19], vertex operator algebras and conformal field theory [19–21].

Abstractly, a complex fusion category is a category that is $\mathbb {C}$-linear, Abelian, finite, semi-simple, monoidal (with unit $\textbf{1}$ and product ⊗ that is bilinear on morphisms), rigid, indecomposable, and for which $\textrm{End}(1) \cong \mathbb {C}$. While this categorical definition is quite involved, these categories can be equivalently described via finite lists of complex numbers: a fusion system. In [22], the exact relation between the abstract notion of a fusion category and the concrete notion of a fusion system is worked out in full detail.

There are several very important extra structures that one can put on a fusion category, or fusion system, like

- A *pivotal structure* that relates an object to its double dual. Concretely, this is a list of *r* complex phases.
- A *braiding*, that provides a means of swapping the order of objects in a tensor product. Concretely, this is a finite set of matrices $\{R^{a,b}_{c}\}$ satisfying a set of polynomial equations.

Depending on the availability and properties of these structures, fusion categories are given different adjectives and find different applications. For example, a braided fusion category provides representations of the braid group [10, 23]. If that category also has a ‘*spherical*’ pivotal structure, then it comes with a quantum trace that allows one to calculate invariants of framed links. If that category is moreover unitary and modular, then it can be used to model systems of anyons: particles confined to a two-dimensional surface whose exchange is modeled by a representation of the braid group [4, 6].

Despite their wide range of applications and the existence of concrete data structures to represent them, it is challenging to obtain an overview of which fusion categories are known and what their properties are. Many available examples of fusion categories are non-explicit, spread throughout the literature, and written down from a vast number of different perspectives. This is an obvious consequence of the wide applicability of fusion categories, but provides frustration to researchers who need explicit data on fusion categories. In this paper we tackle this problem partially by presenting a free package, Anyonica [24], that contains algorithms to find complex multiplicity-free fusion categories of low rank.

All categorifiable rings up to rank 6 have already been classified in [25]. By using Anyonica, we provide a census of pivotal multiplicity-free fusion categories up to rank 7, including pivotal and braided structures. If the $\text {PSU}(2)_{12}$ fusion ring only has pivotal categories then all fusion categories in the census are pivotal. The census would then encompass all multiplicity-free categorifiable fusion rings up to rank 7, along with their pivotal and braided structures. All *F*-symbols, *R*-symbols, pivotal coefficients, and modular data of the fusion categories have been found and are now available as well. The data that describes the categories is symbolic, i.e., no floating-point numbers are used. These symbolic values, as well as a multitude of functions for working with fusion rings and categories, can be accessed as part of the Anyonica package. Their numerical values are freely accessible on a website called the anyonwiki [26].

The reason we use the word census, rather than a classification, is that our results rely on computer algebra in combination with quite a bit of manual bookkeeping of the obtained data. Since the Anyonica package is quite extensive and its evaluation relies on Mathematica (which is closed-source), it is always possible that the results may contain errors due to silent bugs. For the census to be a classification, it needs to satisfy three properties.

1. Correctness. The data in the census represents what it claims to represent: pivotal and possibly braided fusion categories.
2. Completeness. The census contains all multiplicity-free fusion categories up to rank 7 together with all compatible braided and pivotal structures.
3. Uniqueness. None of the categories are equivalent.

We will briefly elaborate on these three properties in Sect. 5, referring the interested reader to [27] for a more in-depth review. Although we won’t describe the result as an actual classification, we do believe that the data obtained is valuable. In particular, we believe the census provides a good starting point for testing hypotheses and finding (counter)examples.

This paper is organized as follows. In Sect. 2, we introduce the notion of a pivotal, spherical, braided, ribbon, and modular fusion system and discuss unitarity of fusion categories. In Sect. 3, we propose a strategy to find multiplicity-free fusion categories of low rank. Then, in Sect. 4, we explain the algorithms we used to implement this strategy and show, by examples, their implementation in Anyonica. In Sect. 5, we concisely present the results of the census of all pivotal and multiplicity-free fusion categories up to rank 7 along with all possible braided structures. We make several general observations and provide diagrams that describe the results. We also discuss the current evidence for the correctness, completeness, and uniqueness of the obtained data.

The appendix contains more info on other software that Anyonica was inspired by and also contains references to other useful software to work with fusion rings and categories.

## Fusion Systems

We start this section by presenting the definition of a fusion system. Then we will add extra structures to arrive at the notions of pivotal, spherical, braided, ribbon, and modular fusion systems. The notion of unitarity of fusion systems and the link with unitarity of its data is discussed as well. Most of the material in this section comes straight from [22], an excellent resource for people who want to bridge the gap between the abstract fusion categories and the down-to-earth numerical fusion systems.

### Definition 2.1

- A **fusion system** is a collection of data $( \textbf{L},*,\textbf{N}, \textbf{F})$ where
  1. $$\textbf{L} = \{ 1, \ldots , r\}$$, $$r \in \mathbb {N}$$.
  2. $*:\textbf{L} \rightarrow \textbf{L}$ is a map for which $1^* = 1$ and $(a^* )^* = a$.
  is a finite set of natural numbers that satisfy 123
  is a finite set of finite-dimensional invertible matrices, with inverses , that satisfy 45678 where whenever a zero dimensional matrix is encountered in a term, it is automatically 0.
- Equations (6) are called the **triangle equations** while equations (8) are called the **pentagon equations**.
- The matrices  are called *F*-matrices, their elements are called *F*-symbols and if a=1 or b=1 or c=1 then we call such the *F*-matrix resp. *F*-symbol a vacuum *F*-matrix resp. vacuum *F*-symbol.

### Remark 2.2

We did not demand that , i.e., that fusion is commutative. Neither did we demand that the *F*-matrices are unitary, as is often assumed in physics literature.

Fusion systems are closely related to fusion categories. In [22] it is shown that each fusion system gives rise to a unique fusion category. On the other hand, each fusion category gives rise to an infinite number of fusion systems. There are two reasons for this phenomenon.

1. On the one hand, a choice of bases, also called a choice of the gauge, is required to determine the *F*-matrices. From a categorical perspective the *F*-matrices correspond to the pull-back of the associator $\alpha : a\otimes (b\otimes c) \rightarrow (a \otimes b) \otimes c$ as a map on objects to a map on the (finite dimensional) morphism spaces. In particular  where $V^{a,b}_c = \hom ( a \otimes b, c )$. Let the matrices , with inverses , represent basis transformations (also called gauge transformations) of the various $V^{a,b}_c$. The induced transformations on the *F*-matrices are of the form 9 For any solution  to the pentagon equations, 10 is also a solution to the pentagon equations. So as long as the gauge transforms are chosen such that (4) (5), (6) and (7) are satisfied for the new set of *F*-symbols (which is always possible), we obtain a fusion system that corresponds to the same fusion category.
2. On the other hand, any permutation σ of the elements of $\textbf{L}$ in such a way that  leaves the pentagon equations invariant and thus for any solution  to the pentagon equations, 11 is also a solution to the pentagon equations. In this paper, we will not refer to such permutations as gauge transforms.

### Remark 2.3

In contrast with [22], definition 2.1 demands that any vacuum *F*-matrix is the identity. Out of the three demands (5), (6), and (7) only the triangle equations, i.e., (6) are necessary. It is however, without loss of generality, always possible to satisfy demands (5) and (7) (see proposition 4.7 for a proof for the multiplicity-free case). The main difference between the triangle equations and demands (5) and (7) is that the former is a necessary consistency condition while the latter two are unnecessary and only there to simplify calculations. The latter two also restrict the choice of gauge, while the first does not.

A fusion system is called **multiplicity-free** if . For a multiplicity-free fusion system, we use the notation  and the demands on the *F*-matrices simplify to121314For a multiplicity-free fusion system each fusion and splitting space is one-dimensional and, by writing , the *F*-symbols transform as15The terms fusion system and fusion category will often be used interchangeably in what follows. The latter is the more common term, while the former is, as far as we are aware, only introduced in [22] to point out the relationship between the abstract structure and the more down-to-earth, numerical description of a fusion category. For most of the arguments in this paper, understanding the category theory is unnecessary. Some arguments do stem from a categorical approach, however. If this is the case, we will give useful references but we will not go into much detail.

### From Fusion System to Modular Fusion System

We start by adding a *pivotal structure*.

#### Definition 2.4

- A **pivotal fusion system** $( \textbf{L}, *, \textbf{N}, \textbf{F}, \textbf{P} )$ is a fusion system together with a list of phases $\textbf{P} = \{ p_a \in \textrm{U}(1)| a \in \textbf{L} \}$, called **pivotal coeffictients**, for which
  16 $$\begin{aligned} p_1= &   1, \end{aligned}$$
  17 $$\begin{aligned} p_a= &   p_{a^*}^{-1}, \end{aligned}$$
  18 for all .
- The **left quantum dimensions** $\{d_a^L \in \mathbb {C}| a \in L \}$ of a pivotal fusion system are defined as 19
- The pivotal structure $\textbf{P}$ is called **spherical** if $d_a^L = d^L_{a^*}$ for all $a \in \textbf{L}$. In this case, the pivotal fusion system is called a **spherical fusion system**, the left quantum dimensions are called the **quantum dimensions** of the system and are written down as $d_a$ rather than $d_a^L$.

#### Remark 2.5

- A pivotal structure is often not regarded as an extra structure in the physics literature. This is because physicists most often work with a unitary fusion system which comes with a unique canonical pivotal structure. We will expand more unitary fusion systems in Sect. 2.2.
- Some authors will refer to the left quantum dimensions as right quantum dimensions. This is only a matter of convention.

The pivotal coefficients are also gauge-dependent. A gauge-transform, with matrices , has the following effect20where the last equality comes from the fact that we do not allow changes in the vacuum *F*-symbols, and thus . The reason this is the correct transform comes from a categorical argument. For a pivotal fusion category, the left quantum dimensions $d^L_a$ are defined in a basis-independent manner and must therefore be gauge-invariant. Since the left quantum dimensions are gauge-invariant, $p_a$ must transform the same way as , i.e., as in Eq. (20).

We can also add a *braided structure* to a fusion system. This structure is independent of a pivotal structure.

#### Definition 2.6

- A **braided fusion system** $( \textbf{L}, *, \textbf{N}, \textbf{F}, \textbf{R} )$ is a fusion system together with a finite list $\textbf{R}$ of invertible matrices $\Big \{\left. \big [R_{c}^{ab}\big ] \in \text {Mat}_{N^{a,b}_{c} \times N^{a,b}_{c}}(\mathbb {C}) \right| a,b,c = 1,\ldots ,r \Big \}$, with inverses , that satisfy the hexagon equations: 2122
- A **ribbon fusion system** is a spherical braided fusion system.
- A **modular fusion system** $( \textbf{L}, *, \textbf{N}, \textbf{F}, \textbf{P}, \textbf{R} )$ is a ribbon fusion system for which the matrix $\hat{S} \in \text {Mat}_{r \times r}(\mathbb {C})$, whose entries are given by 23 is invertible.

In [22] it is shown that each of the systems above defines a fusion category, with the corresponding adjectives, that is unique up to equivalence.

Just like the *F*-symbols and pivotal coefficients, the *R*-symbols are gauge-dependent. A gauge-transform, with matrices , has the following effect24If the underlying fusion system is multiplicity-free, then several formulas are simplified. The pivotal equations lose the summation on the RHS and are simplified to25If we let , then the hexagon equations simplify to2627and the matrix $\hat{S} \in \text {Mat}_{r \times r}(\mathbb {C})$, has entries28For the multiplicity-free case, the gauge transforms (20) and (24) also simplify to2930

#### Remark 2.7

- The origin of the Eqs. (16), (17) and (18) is described in [22]. They stem from the fact that $$x^{**}\cong x$$ in the categorical language rather than $$x^{**}=x$$. For several formulas, including the one to calculate left quantum dimensions, a set of isomorphisms $$\psi _x:x^{**} \rightarrow x$$ is required to match the input and output of various morphisms properly. Requiring that a given set of isomorphisms $$\{\psi _x\}$$ behaves naturally with respect to the tensor product comes down to demanding the pivotal equations.
- For each ribbon fusion system, there exists a canonical list of gauge-independent phases, also called twists, $\textbf{T} = \{ \theta _i \in \text {U}(1)| i \in \textbf{L} \}$ that satisfy equations
  31 $$\begin{aligned} \theta _{1}= &   1, \end{aligned}$$
  32 $$\begin{aligned} \theta _{a}= &   \theta _{a^*}, \end{aligned}$$
  3334 or in the case of a multiplicity-free category:
  35 $$\begin{aligned} \theta _{1}= &   1, \end{aligned}$$
  36 $$\begin{aligned} \theta _{a}= &   \theta _{a^*}, \end{aligned}$$
  3738
- The *S*-matrix of a modular fusion system is defined as
  39 $$\begin{aligned} [S]^{a}_{b} = d_{a} d_{b} [\hat{S} ]^a_{b}. \end{aligned}$$
  It can be shown that it is, up to scaling, always a unitary matrix [28]. The rank of *S* equals that of $\hat{S}$, but typically, $\hat{S}$ has a nicer form. Moreover, $\hat{S}$ can be defined independent of the specific pivotal structure, so to calculate the *S*-matrix for multiple pivotal fusion systems with the same *F*-and *R*-symbols, one only has to calculate $\hat{S}$ once.
- Let *T* be an r×r matrix with entries
  40 $$\begin{aligned} [T]^a_{b}:= \delta ^a_b \theta _{a}, \end{aligned}$$
  then, for a modular fusion system, *S* and *T* satisfy
  41 $$\begin{aligned} (ST)^3 = \lambda S^2, \end{aligned}$$
  42 $$\begin{aligned} S^4 = \mathbb {1} \end{aligned}$$
  for some $\lambda \in \mathbb {C}^{\times }$ [1]. They define a projective representation of the modular group $\text {SL}_{2}(\mathbb {Z})$, hence the origin of the adjective ‘modular’.
- Via a construction known as the center of a category, one can construct modular fusion categories from spherical fusion categories [29]. This is one of the good reasons why it is interesting to study more general fusion categories than just modular ones.

### Unitary Fusion Systems

While in the physics literature unitarity is often taken for granted, we decided not to do so. Not demanding unitarity from the start makes it easier to see several subtleties that were otherwise hidden. One of these subtleties is the existence (and choice) of different pivotal and possibly non-spherical structures. Categorically a unitary fusion category is a fusion category that comes with a *Hermitian structure*, †, that satisfies several properties (see, e.g., [30, 31] for the full definition). The Hermitian structure defines the notion of unitary maps and can, in particular, be used to define evaluation and coevaluation morphisms that can be combined to calculate the left quantum dimensions $d^L_a$ [32–34]. The left quantum dimensions, arising this way, satisfy $d^L_a = d^L_{a^*} = \textrm{FPDim}(a)$ where $\textrm{FPDim}(a)$ is the largest real eigenvalue of the matrix $[N_a]$ with entries $[N_a]^{c}_b = N_{a,b}^c$. Due to the Frobenius-Perron theorem this value is unique and $\textrm{FPDim}(a) \ge 1$ for all $a \in \textbf{L}$. The Hermitian structure also fixes a spherical pivotal structure $\textbf{P}$, which, for the corresponding fusion system, is given by . This spherical structure is also called the canonical spherical structure of the unitary fusion category.

While a priori, it looks like there might be multiple Hermitian structures that one can put on a fusion category, it was recently shown in [35] that any unitarizable fusion category admits a unique unitary structure (up to unitary monoidal equivalence). Therefore, the canonical spherical structure can be implicitly included in the definition of a unitary fusion category.

In [33] (part 4) it is shown that if a fusion system has a gauge for which its *F*-matrices are unitary, then its category can always be made into a unitary category via the choice of an appropriate Hermitian structure. Therefore we define a unitary fusion system as follows

#### Definition 2.8

A **unitary fusion system** is a spherical fusion system $(\textbf{L},*,\textbf{N},\textbf{F},\textbf{P})$ for which the *F*-matrices are unitary and the spherical structure is such that the quantum dimensions satisfy $d_a = \textrm{FPDim}(a)$.

For a unitary category, there always exists a gauge choice for which the *F*-symbols of the corresponding spherical fusion system are unitary. So, the notion of a unitary fusion system is equivalent to that of a unitary fusion category.

It is important to note that if the *F*-matrices of a fusion system are unitary, one could still choose a (spherical) pivotal structure that is not canonical. For example, any fusion system with a fusion ring corresponding to the group ring $\mathbb {Z}_3$ has unitary *F*-symbols, but there are multiple non-spherical pivotal structures for which the quantum dimensions are not positive. We will not call these fusion systems unitary, but rather say that they admit a unitary gauge.

The definition of unitarity of a fusion category can be extended to braided, ribbon, and modular fusion categories by demanding that the braided and ribbon structures are *compatible with the unitary structure*. In the paper [30], these demands are written out, and the following statements are proven:

- If a unitary fusion category admits a braiding, it is automatically a unitary braided fusion category.
- Every unitary braided fusion category admits a unique unitary ribbon structure. This ribbon structure is the canonical ribbon structure that comes from the canonical spherical structure in combination with the braiding.

In particular, a unitary braided fusion category is immediately a ribbon fusion category. We will therefore use the following definitions

#### Definition 2.9

- A **unitary braided fusion system**, or equivalently a **unitary ribbon fusion system**, is a ribbon fusion system $( \textbf{L}, *, \textbf{N}, \textbf{F}, \textbf{P}, \textbf{R} )$ for which $( \textbf{L}, *, \textbf{N}, \textbf{F}, \textbf{P} )$ form a unitary fusion system and the *R*-matrices are unitary matrices.
- A **unitary modular fusion system** is a unitary ribbon fusion system for which the matrix $\hat{S}$ is invertible.

We end this chapter by noting that not all fusion categories are unitary. Typically, the non-unitary categories lead to a much richer variety of examples. This is because the cascade of canonical structures that follow from the Hermitian structure of a unitary category is not there for a generic category. Therefore, one can put many more combinations of different structures on non-unitary categories.

#### Remark 2.10

The demand that the *F*-and *R*-matrices of a unitary fusion system are unitary is out of convenience rather than necessity. Since such a basis always exists and it is not hard to find (see Sect. 4.1.9 for an algorithm) this should cause no issues in practice.

## The Landscape of Fusion Categories

Every fusion category is fully determined by a fusion system $(\textbf{L},*,\textbf{N},\textbf{F})$. The mathematical structure that captures the properties of $\textbf{L}, *$, and $\textbf{N}$ is that of a fusion ring [1], which is defined as follows.

### Definition 3.1

A $\mathbb {Z}_+$-ring is an associative unital ring $\mathcal {R}\equiv (\mathcal {R},B,1,\times ,+)$, finitely generated and free as a $\mathbb {Z}$-module, which is equipped with a distinguished basis *B* such that 1∈B and for which the structure contants $\{ N_{ab}^c \}$ are non-negative.

A **fusion ring** $\mathcal {R} \equiv (\mathcal {R},B,1,\times ,+,*)$ is a $\mathbb {Z}_+$ ring with basis *B* and a linear involution $\cdot ^*:a \mapsto a^*$ such that  and  for all a,b,c∈B. The size of *B* is called the **rank of the fusion ring**, and the number  is called the **multiplicity of the fusion ring**. If the multiplicity of a fusion ring is 1 we say the ring is **multiplicity-free**.

If the Frobenius-Perron dimensions of the elements of the fusion ring, i.e. the largest eigenvalues of the matrices $N_a$ with , are integers we say the fusion ring is **integral**. If a fusion system (or category) has an integral fusion ring we say the fusion system (or category) is integral.

### Remark 3.2

Since a fusion ring is entirely determined by a finite set of integer structure constants , there are only a finite number of fusion rings for a given rank *r* and multiplicity *m*.

A fusion ring is the only required information to set up the necessary constraints (4), (5), (6), (7), and (8) on the *F*-symbols in order to obtain a fusion system. These constraints form a system of polynomial equations and inequalities. Such a system is, in theory, always algorithmically solvable, but it could be that the solution set is empty. Solving these equations is also known as categorifying the fusion ring.

### Classification of fusion categories

Most known fusion categories are of some ‘*standard form*’, a combination of these (via, e.g., tensor products), modifications of these (via, e.g., zesting [36]) or extensions of these (via, e.g., constructions such as the Tambara-Yamagami [37] or Haagerup-Izumi [38] categories). By a *standard form* we^1^ mean that the fusion ring is one of the following:

- A group ring of a finite group.
- A ring of finite dimensional irreducible representations of a finite group with the product being the tensor product.
- A fusion ring coming from the representation theory of quantum groups at roots of unity (see [39] for a gentle introduction).

To what extent constructions using these standard categories populate the landscape of fusion categories is unknown. There are several directions in which classification results exist.

- One direction targets categories by dimension^2^, either via constraints on the dimension of the category, e.g., [40, 41] or via constraints on the dimensions of some of the objects, e.g., [42, 43].
- Another direction is where the rank of a fusion ring is fixed and categories of that rank are classified for all multiplicities. This approach has, so far, only been successful for classifying rank 2 fusion categories [44] and rank 3 pivotal fusion categories [45].
- The final direction we would like to mention is the one this paper takes. If one fixes the multiplicity, in our case to 1, there are only a finite number of fusion categories per rank and one can try to classify fusion categories rank by rank.

The classification directions described above are not suitable for the ambitious goal of a full classification (just like finite groups are also not classified by order). Since only a few examples of fusion categories are known, partial classifications are still of tremendous importance to advance research in the area of fusion categories.

It is important to note that a lot of fruitful work has been done on the classification of fusion categories with specific properties. For results on fusion categories related to quantum groups see, e.g., [46–49]. For so called *near-group categories*, such as the Tambara-Yamagami and Haagerup-Izumi categories, see, e.g., [37, 50, 51]. Recently, further improvements on the classification of so called *G*-*quadratic* fusion categories up to rank 4 (with multiplicity) were made in [52]. The classification of braided categories has been a central topic in, e.g., [53, 54] and appears as a special case of [55] where it was shown that there are only a finite number of braided fusion categories per rank (which improves upon the earlier result that there are only a finite number of modular categories per rank [56]). The classification of ribbon categories up to rank 4 has been investigated in [57] and the special case of *pseudo-unitary* ribbon fusion categories of rank 5 has been investigated in [58]. There are so many results on the classification of modular categories that we cannot do justice to all of them. Some of the strongest and most general results are the following. All modular data, i.e. *S*-matrices and *T*-matrices, for fusion categories up to rank 12 has been classified in [59] and modular data of integral fusion categories has been classified up to rank 13 in [60]. For classification results of modular categories based on dimension see, e.g., [61–66].

#### Remark 3.3

Classification results can have a variety of levels of abstraction. Some authors classify fusion categories with certain properties by creating bijections with other *simpler* algebraic structures. Other authors claim all fusion categories with certain properties are classified once the categorifiable fusion rings that lead to such categories are classified. In this paper we use a more explicit interpretation of the word ‘classification’. We say that all fusion categories with certain properties are classified if all data on their fusion systems (i.e. their *F*-symbols, *R*-symbols, and pivotal coefficients) is available in a computational format. Moreover this format must give an exact representation of the fusion categories, which in our case is achieved by representing all symbols as elements of finite field extensions of $\mathbb {Q}$.

### Classifying fusion categories by solving consistency equations

In order to get an overview of a small part of the landscape of fusion categories one could, theoretically, apply the following approach:

1. Fix a multiplicity *m*, and a rank *r*.
2. Find all fusion rings with this rank and multiplicity.
3. Solve the pentagon equations per fusion ring found.
4. Solve the pivotal and hexagon equations per solution to the pentagon equations.
5. For each triple of *F*-symbols, *R*-symbols (possibly none), and pivotal coefficients (possibly none but not for any of the categories we considered), calculate all other properties such as the quantum dimensions, twists, and the *S*-matrix.

**Fig. 1:**
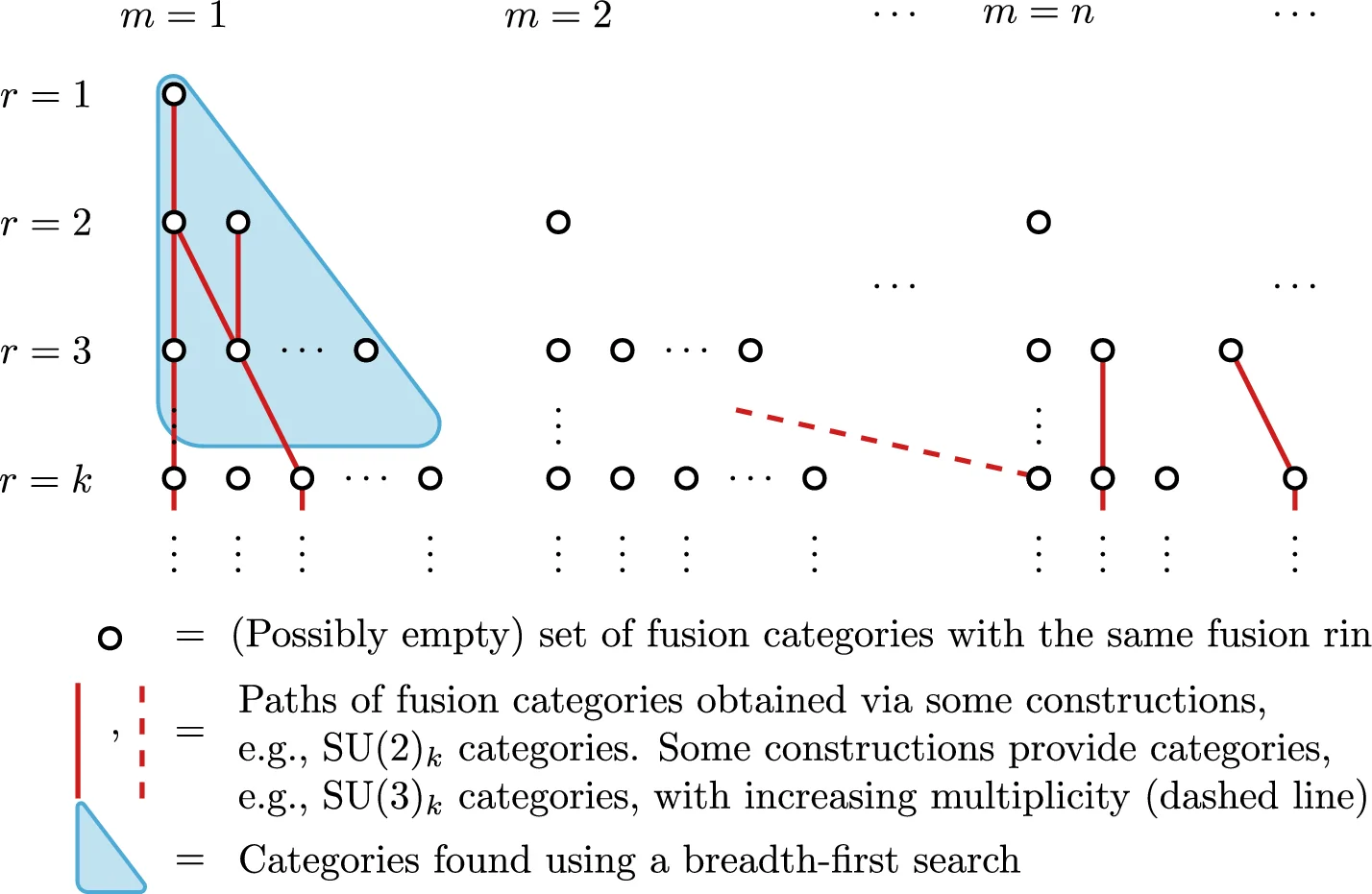
By fixing a multiplicity *m* and listing fusion rings by rank *r*, one can visualize the landscape of fusion categories as a series of tables. While the standard constructions cut out (infinitely) deep paths in these tables, our approach cuts out a wide area in the m=1 table

If one fixes the multiplicity *m* and lists fusion rings by their rank *r*, one gets a table of possible fusion categories per fixed value of *m*. While the standard constructions typically provide several categories for each rank *r* and thus descend deep into these tables, the approach above produces all categories per rank, i.e., it goes wide into the table (see Fig. 1).

There are several caveats when applying this strategy, however.

**Fig. 2:**
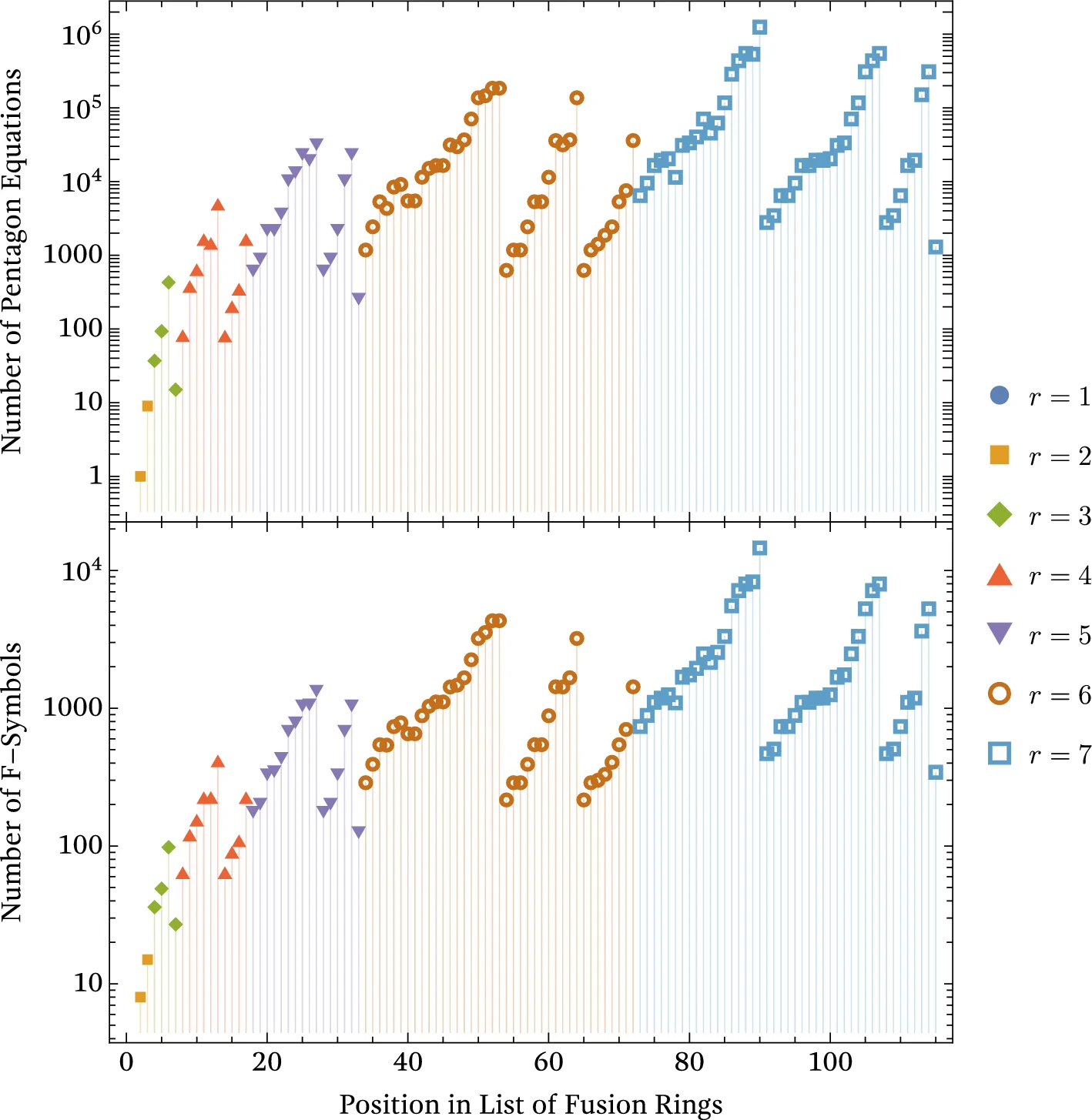
Number of pentagon equations and *F*-symbols per fusion ring. The numbers on the horizontal axis correspond to the position of the fusion ring in the list of multiplicity-free fusion rings on the anyonwiki and in the FusionRingList in Anyonica. Here, we assumed that the vacuum *F*-symbols are already known and the trivial pentagon equations have already been removed. This, and the fact we use a logarithmic scale, is also why the trivial ring at position 1 has no data

1. Firstly, due to gauge symmetry, there are an infinite number of solutions to the consistency equations that, in essence, describe the same fusion system. Without breaking this gauge symmetry beforehand, solving the consistency equations is only possible for the simplest examples. For fusion rings with multiplicity, i.e., with  for some labels, we don’t know of a general way to break this symmetry. If the fusion ring is multiplicity-free, however, the symmetry transformations have a simple form, and we can get rid of this symmetry before solving the pentagon equations. We, therefore, decided to restrict our attention to the categorification of multiplicity-free fusion rings.
2. Secondly, while theoretically, any set of polynomial equations can be solved algorithmically, there are some serious limits on the systems for which this can be done in practice. A system of polynomial equations is algorithmically solved by finding a suitable Gröbner basis that brings the system of equations into an “upper triangular” form. By this, we mean a new system with the same roots as the original one, but from which one can find all roots by only solving polynomial equations in one variable. Most computer algebra systems have standard built-in algorithms to calculate Gröbner bases, such as Buchberger’s original algorithm [67], or the more recent and more optimized F4 [68] and F5 [69] algorithms. We found that none of these algorithms is capable of solving the pentagon equations, without some serious pre-processing of the system. The number of equations and *F*-symbols just grows too fast with the rank of the fusion ring (see Fig. 2 for the exact numbers).

By using methods specifically designed for solving pentagon and hexagon equations, we found all multiplicity-free pivotal fusion categories up to rank seven, however. Much of the progress in solving these equations comes from methods and heuristics aimed at reducing sparse systems of polynomial equations. While these methods were written to solve pentagon and hexagon equations, it soon became clear that they are more widely applicable. Therefore we decided to create the Anyonica package [24].

Anyonica has multiple use cases. For one, it is meant to ease research in the theory of anyon models and, more generally, fusion categories. To this end, it contains lists of fusion rings and categories together with all the relevant data we could find. Moreover, many functions have been implemented to aid in finding properties of fusion rings and categories and transforming and or combining them. The second way in which the package is useful is via the many specialized functions it provides for reducing large systems of polynomial equations. Different reduction techniques and heuristics can and have been applied for solving both the pentagon and hexagon equations as well as for other systems of polynomial equations, such as the equations governing anyons on wire networks [70]. The package, written in the Wolfram Language, a.k.a. Mathematica, is freely available on https://github.com/gert-vercleyen/Anyonica.

In the next sections we will explain the various algorithms developed and how they helped find fusion categories. Also, small examples of code will be provided to illustrate how the Anyonica package can be used. Most of the examples will focus on the fusion ring $\text {FR}^{3,1,0}_{2}= \text {Rep}(D_3)$ (where $\text {FR}^{3,1,0}_{2}$ is the formal name used on the anyonwiki [26]) which has the following fusion rules

$$ \begin{array}{|lll|} \hline \textbf{1} &  \textbf{2} &  \textbf{3} \\ \textbf{2} &  \textbf{1} &  \textbf{3} \\ \textbf{3} &  \textbf{3} &  \textbf{1}+\textbf{2}+\textbf{3} \\ \hline \end{array}. $$

These examples should be regarded as if they belong to the same Mathematica session, so if the variable repD3 is defined in one example, it must be assumed that it is accessible in the other examples as well. Although the programming style and keywords are those of the Wolfram Language, the code should be as readable as any pseudo-code.

#### Remark 3.4

- There is no reason to believe that there won’t be a generic algorithm that can tackle the pentagon equations sometime in the future. Often, a direct Gröbner basis calculation is thought to be undoable since theoretically (see [71]) the largest degree of an element of a Gröbner basis is bounded above by
  43 $$\begin{aligned} 2 \left( \frac{d^2}{2} + d \right) ^{2^{n-2}}, \end{aligned}$$
  with *d* the highest degree of the input polynomials (always 3 in our case), and *n* the number of variables (easily larger than 100 for fusion rings of rank 5 or higher as can be seen in Fig. 2). While there exist polynomial systems for which such a degree is obtained [72], we found that the pentagon equations typically can be reduced to a Gröbner basis of a degree of the order of *r*. The double exponential asymptotic scaling in time and memory of Gröbner basis algorithms might be correct for arbitrary systems of polynomial equations but the pentagon and hexagon equations are in no way arbitrary. There is a lot of structure behind these equations that is not taken into account in an expression for a bound like (43)
- Some other efforts at achieving similar results have also been made. These will be reviewed in Sect. A.

## Categorifying Fusion Rings: Solving Consistency Equations

In [73] we described the methods we used to find low rank fusion rings. The search resulted in a complete classification of multiplicity-free fusion rings up to rank 9. We will assume from now on that the data of any multiplicity-free fusion ring up to rank 7 is known. The workflow of categorifying a fusion ring is the following

1. First, we set up the pentagon equations and combine these with the constraints that each *F*-matrix must be invertible, i.e., $\Big \{\left. \det ([F_{d}^{abc}]) \ne 0\right| a,b,c,d = 1, \ldots , r\text { for which }N_{a,b,c}^d \ne 0\Big \}$. The other constraints, (13), on the *F*-matrices can be implemented beforehand to reduce the number of variables. The consistency relations are then solved to obtain sets of *F*-matrices. Next, the sets of *F*-matrices for which a basis exists in which they are unitary are expressed in such a basis. Some of the sets of *F*-symbols might be equivalent due to a gauge transform, possibly in combination with a fusion ring automorphism. By removing redundant solutions, we obtain a final set of inequivalent fusion systems, say $\left\{ \mathcal {F}_i:= ( \textbf{L},*,\textbf{N}, \textbf{F}_i) \right\} _{i=1}^{n_F}$.
2. For each fusion system, the hexagon equations, (26) and (27), are set up and combined with the constraints that none of the *R*-symbols can be 0. If there are solutions to the hexagon equations, then these are combined with their respective fusion system into a braided fusion system. Here, we also might have equivalent braided fusion systems, and the redundant ones are removed as well. The result is a set of possibly braided fusion systems $\left\{ (\mathcal {F}_i, \mathcal {R}_{i,j} ) \right\} _{j=1}^{n_R(i)}$ where $\mathcal {R}_{i,j}$ might be empty in the case of a non-braided fusion system.
3. For each braided fusion system, the pivotal constraints, (25), are solved. The solutions to these equations are combined with the (braided) fusion systems. Here, we also might have equivalent (braided) pivotal fusion systems, and redundant systems have to be removed as well. The result is a set of (braided) pivotal fusion systems $\left\{ (\mathcal {F}_i, \mathcal {R}_{i,j}, \mathcal {P}_{i,k} ) \right\} _{k=1}^{n_P(i)}$ where $\mathcal {R}_{i,j}$ might be empty. Although it is a famous open question whether the pivotal equations always have a solution, this is the case for all the fusion systems we investigated.

We will, despite taking other constraints into account, from now on refer to step 1 as “solving the pentagon equations”, step 2 as “solving the hexagon equations” and step 3 as “solving the pivotal equations”.

In order to solve these equations, several techniques must be applied to reduce the systems of consistency equations. This section aims to explain the various methods used to reduce and eventually solve these sets of consistency equations. In particular, Sect. 4.1 explains the various techniques developed and their application to solving pentagon equations. Most, but not all, of the techniques used also find their application in solving the hexagon equations. The minor differences between the strategy used to solve the hexagon equations and that to solve the pentagon equations are explained in Sect. 4.2. Section 4.3 explains how the pivotal equations were solved.

### Remark 4.1

Steps 2 and 3 in the process of finding fusion categories are independent and can be interchanged.

### Solving pentagon equations

This section explains the procedures for solving the pentagon equations in the order they are applied when one evaluates the function SolvePentagonEquations from the Anyonica package. The only procedures not a standard part of the SolvePentagonEquations function are checking for obstructions to categorification, trying to fix a unitary gauge, and removing duplicate solutions. Checking obstructions was done manually since the SageMath software package is necessary for some criteria.

#### Obstructions to categorification

The quickest way to categorify a fusion ring is not categorifying it at all. Before starting the process of categorification, it is interesting to check whether there might be obstructions from categorification. The criteria used all come from the papers [25, 74], and [75]. Table 1 gives an overview of the various criteria.

**Table 1:** The various categorifiability criteria that were used. Here “Only Commutative” means that the criterium only applies to commutative fusion rings. FC, UFC, and PFC respectively stand for fusion category, unitary fusion category, and pivotal fusion category

Criterion	Abbreviation	Only commutative	Rules out
Zero-spectrum	ZS	No	FC
Commutative schur product	CSP	Yes	UFC
*d*-number	DN	Yes	FC
Pivotal drinfeld center	PDC	Yes	PFC
Lagrange	L	No	FC
Extended cyclotomic	EC	Yes	FC

While we focus on categorifying multiplicity-free fusion rings, some of these criteria were easy enough to apply to rings with multiplicity as well.

The ZS criterion is implemented in Anyonica as ZSCriterion and was able to rule out general categorification for 267 of the 28451 fusion rings stored in Anyonica.

The CSP criterion is implemented via the function CSPCValue, whose output is a floating point number *p*. If p<0, then the fusion ring has no unitary fusion category. We could not implement an exact test since the CSP criterion uses fusion ring characters, and we do not have symbolic versions of all character tables. This is also why a number is returned rather than a boolean: it is up to the user to determine whether |*p*| is big enough to be convinced that the test result is correct. For an accuracy of 64 decimal digits, the CSP criterion ruled out unitary categorification for 19471 of the 28333 commutative fusion rings stored in Anyonica.

Some of the more decisive criteria, such as the DN, PDC, L, and EC criteria, require algorithms from number field theory (see [25] for the source code). These algorithms could be highly demanding, to the point of needing more than 128GB of RAM. These were, therefore, only applied to the multiplicity-free fusion rings, some of which were still too hard to crack. The DN, PD, and L criteria are implemented in Anyonica as DNCriterion, PDCCriterion, and LCriterion. Mathematica does not have the required functionality that is needed to implement the EC criterion, so we used the SageMath code from the original paper to calculate its outcome.

##### Example 4.2

The following code

**Figure:**
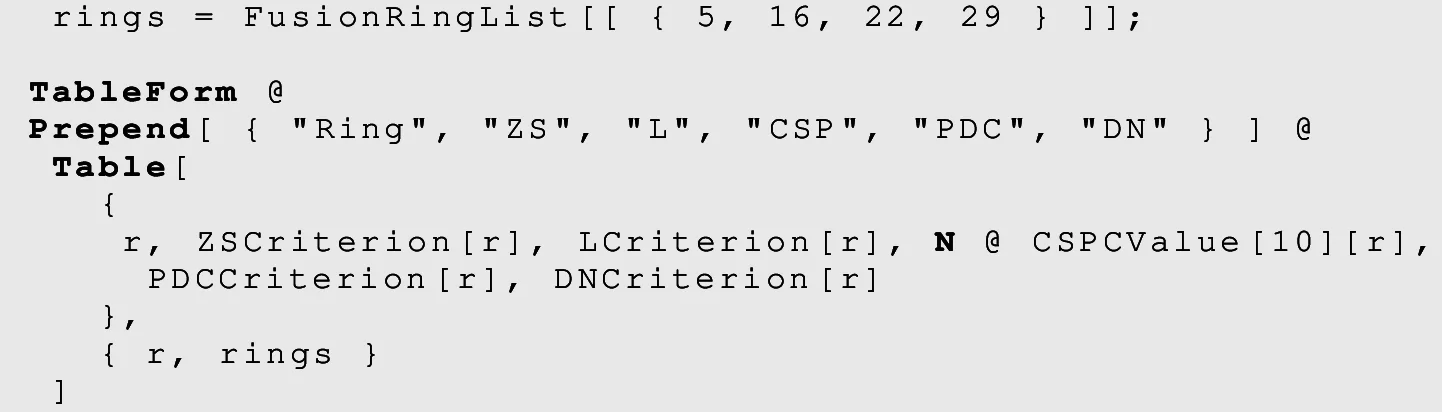

returns

**Figure:**
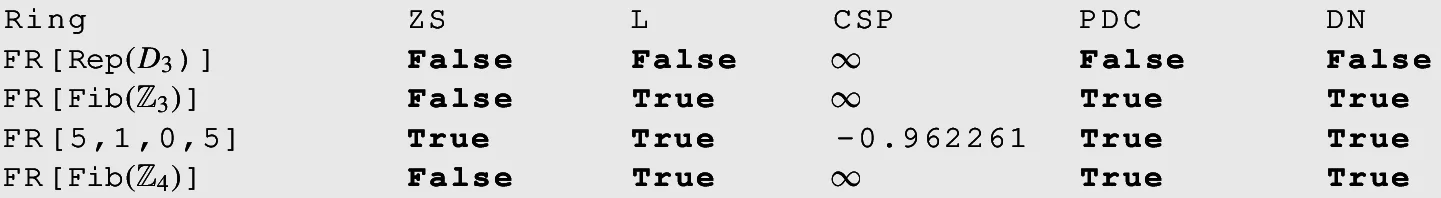

So for the first ring, $\text {Rep}(D_3)$, none of the criteria exclude categorification, while for each of the other rings, there is at least one criterion that rules out categorification. The numerical value, different from ∞, indicates that the third ring has no unitary categorifications due to the CSP criterion. The other criteria already made the stronger statement that this ring has no categorifications at all, however. As a matter of fact, all multiplicity-free fusion rings up to rank 7 for which the CSP criterion rules out unitary categorification are already ruled out from general categorification due to some other criteria.

On the page of small multiplicity-free fusion rings on the anyonwiki, the categorifiability data of the multiplicity-free fusion rings with rank 8 to 9 is listed. All rings of rank 8 or greater, except for $\text {FR}^{8,2}_4$, $\text {FR}^{8,4}_2$, $\text {FR}^{8,4}_5$, $\text {FR}^{9,2}_2,$ and $\text {FR}^{9,6}_3$, that are listed as not categorifiable were ruled out because of the DN, PDC, L, or EC criteria. The rings $\text {FR}^{8,2}_4, \text {FR}^{8,4}_2, \text {FR}^{8,4}_5, \text {FR}^{9,2}_2,$ and $\text {FR}^{9,6}_3$ are ruled out from categorification by using Anyonica to reduce the pentagon equations to the point it was clear that no solution exists. The rings listed as categorifiable, but for which we don’t have explicit data, are rings with a special structure. For example fusion rings arising from finite groups, the representation theory of quantum groups at roots of unity, or products of these are automatically categorifiable.

##### Remark 4.3

There is one fusion ring, $\text {FR}^{9,0}_{38}$, that has no pivotal categorifications due to the PDC criterium but is not ruled out from general categorification by any of the other criteria. This ring is, therefore, the only multiplicity-free fusion ring of rank 9 or less that might be a counterexample to the famous conjecture that all fusion categories have a pivotal structure.

#### Determining which *F*-symbols could be zero

Multiple steps in solving the pentagon equations assume that the variables to be solved for are non-zero. Therefore, we must determine which variables could be zero beforehand. The main steps that we performed, in the order they are applied, are the following:

1. Translate the pentagon equations and invertibility constraints to a logical proposition.
2. Reduce the proposition via tetrahedral symmetries, pattern matching, and conversion between normal forms.
3. Find all lists of booleans that satisfy the proposition.

**Constructing the Logical Proposition.** The pentagon equations and the demands that all *F*-matrices have full rank can be translated to a boolean proposition in the following way. First, every *F*-symbol $F_I$ (where *I* is some admissible label) is replaced by a boolean variable $b_I$ for which

44 $$\begin{aligned} b_I = {\left\{ \begin{array}{ll} \text {FALSE} &  \text { iff } F_I = 0 \\ \text {TRUE} &  \text { iff } F_I \ne 0 \end{array}\right. }. \end{aligned}$$

Next, for every pentagon equation, a proposition is constructed that demands that it is not allowed for exactly one term to be non-zero. For example, for the following equation

45 $$\begin{aligned} F_I F_J = F_K F_L F_M \end{aligned}$$

this translates to the proposition

46 $$\begin{aligned} b_I \wedge b_J \Leftrightarrow b_K \wedge b_L \wedge b_M, \end{aligned}$$

while for

47 $$\begin{aligned} F_{I} F_{J} = F_K F_L F_M + F_N F_O F_P \end{aligned}$$

this translates to

48 $$\begin{aligned} \lnot \left( \left( p_1\wedge \lnot p_2\wedge \lnot p_3\right) \vee \left( \lnot p_1\wedge p_2\wedge \lnot p_3\right) \vee \left( \lnot p_1\wedge \lnot p_2\wedge p_3\right) \right) , \end{aligned}$$

with

49 $$\begin{aligned} p_1 = b_I \wedge b_J, \end{aligned}$$

50 $$\begin{aligned} p_2 = b_K \wedge b_L \wedge b_M, \end{aligned}$$

51 $$\begin{aligned} p_3 = b_N \wedge b_O \wedge b_P. \end{aligned}$$

The demand that the *F*-matrices are invertible would typically translate to the determinant of each matrix being non-zero. There is no way to translate such a demand to a proposition in the variables $b_I$ since it depends on the exact values of the *F*-symbols. Therefore, we weaken this demand to the demand that at least one of the terms of the determinant should be non-zero. If this demand is violated, the determinant is equal to zero, but the opposite is clearly not true.

Let $p(b_{I_1},\ldots ,b_{I_{n}} )$ be the logical conjunction of all propositions constructed for a certain fusion ring. Then, the task of finding all admissible sets of zero values of *F*-symbols comes down to finding lists of booleans for which *p* evaluates to $\text {TRUE}$. This problem is a specific case of an ALLSAT problem in theoretical computer science. However, the proposition *p* is often too big to handle directly, so some heuristics were implemented to reduce its complexity.

**Reducing the Proposition.** Several methods were applied to reduce the proposition *p*. Firstly, one can use so-called tetrahedral symmetries, which allow one to identify several of the boolean variables as follows52These symmetries come from a group of natural isomorphisms in the fusion category (see definition 5.1. in [75]) that map *F*-symbols to other *F*-symbols, up to a non-zero scalar factor. In the physics literature, sometimes a stronger form of these symmetries, namely53is demanded (see [76] for examples and applications). This helps reduce the pentagon equations, but there are fusion systems for which the stricter version (53) does not hold. For example, for non-commutative rings some equalities (coming from mirroring the tetrahedron) demand equality between *F*-symbols that don’t exist and others that do. The equations (52) are only related via *rotations* of the tetrahedron and (at least for the boolean variables) work for any fusion category.

##### Remark 4.4

The factors that appear in front of the *F*-symbols after applying the natural transformations come from the fact that in the categorical language $$x^{**}\cong x$$ rather than $$x^{**}=x$$. The translation to a language where $$x^{**}=x$$ must involve the isomorphisms $$(x^{**}\rightarrow x) \in \hom (x^{**},x)$$ which cause nonzero numerical factors to appear. The group of natural isomorphisms is a group of linear transformations between one-dimensional vector spaces, so they must map non-zero *F*-symbols to non-zero *F*-symbols and vice versa. In particular, they hold exactly on the variables $$b_I$$.

The reduction in the number of variables by using tetrahedral symmetries is quite significant. For example, for the fusion ring $\text {PSU}(2)_6$, the number of variables in the proposition gets reduced from 163 to 44.

Once the tetrahedral symmetries are exhausted, one can still reduce the system by using pattern matching and recursion. The invertibility constraints on the 1*D* *F*-symbols cause *p* to contain sub-propositions of the form (a) $b_{I_1} \wedge \ldots \wedge b_{I_{k}}$, and (b) $b_{I} \Leftrightarrow b_{J}$, that can be factored out. Factors of form (a) can be used to conclude that $b_{I_{1}} = \text {TRUE}, \ldots ,$ and $b_{I_k} = \text {TRUE}$ and this new information can be used to update *p*. Factors of form (b) can be used to set up equivalence relations between certain variables and, therefore, reduce the number of variables appearing in *p*. After updating *p* in this way, new factors of the form (a) and (b) can appear, and the process is repeated until no factors are left. One last procedure can be applied to reduce the proposition. Propositions can be expressed in certain normal forms, such as

- CNF (Conjunctive Normal Form): the proposition is written as an AND of OR’s,
- DNF (Disjunctive Normal Form): the proposition is written as an OR of AND’s.

For example, proposition (46) looks like

54 $$\begin{aligned}&\begin{array}{c} (\lnot F_I\vee \lnot F_J\vee F_K) \wedge (\lnot F_I\vee \lnot F_J\vee F_L)\wedge (\lnot F_I\vee \lnot F_J\vee F_M)\wedge \\ (F_I\vee \lnot F_K\vee \lnot F_L\vee \lnot F_M)\wedge (F_J\vee \lnot F_K\vee \lnot F_L\vee \lnot F_M) \end{array}&\end{aligned}$$

in CNF, but like

55 $$\begin{aligned} \begin{array}{c} (F_I \wedge F_J \wedge F_K \wedge F_L \wedge F_M) \vee (\lnot F_I \wedge \lnot F_K) \vee (\lnot F_I \wedge \lnot F_L) \vee \\ (\lnot F_I \wedge \lnot F_M) \vee (\lnot F_J \wedge \lnot F_K) \vee (\lnot F_J \wedge \lnot F_L) \vee (\lnot F_J \wedge \lnot F_M) \end{array} \end{aligned}$$

in DNF. By expressing the proposition in CNF form, sometimes extra factors of the form (a) can be identified. When the proposition is expressed in DNF form, sometimes variables appear in all AND clauses, which implies they have to be TRUE. The method used to find zero values converts back and forth between CNF and DNF forms to extract these extra pieces of information until no extra info can be extracted.

**Finding all Instances.** Mathematica has a function SatisfiabilityInstances, which can be used to find all boolean vectors that satisfy a proposition. It is also possible to use the built-in function Reduce or our implementation of an ALLSAT solver that writes, compiles, and executes C code without leaving Mathematica. This solver uses the same technique as used in [73] to find fusion matrices.

All of the above functionality has been implemented in the FindZeroValues function of Anyonica. This function takes two arguments equations and variables and several optional arguments. The option "FindZerosBy" allows you to change the algorithm to solve the ALLSAT problem, while "InvertibleMatrices" allows you to add matrices of variables that are required to be invertible. By default, not all equations containing more than two terms are included in the boolean proposition. This is because these equations are often much weaker than those with only two terms. Also, the execution time and memory pressure of the algorithm increases drastically by including all such equations. So by default, FindZeroValues only includes 30% of the *least complex* equations with more than two terms, a value that can be changed via the option "SumSubSetParameter". At the end of the algorithm, however, solutions incompatible with the other 70% of these equations are removed, so this value has no effect on the output itself. The default measure of equation complexity that ZeroValues uses is the built-in LeafCount function, which returns the number of leaves that the formal tree form of an expression has. A custom function can be used by setting the "SumSubsetFunction" option.

##### Example 4.5

The following code

**Figure:**
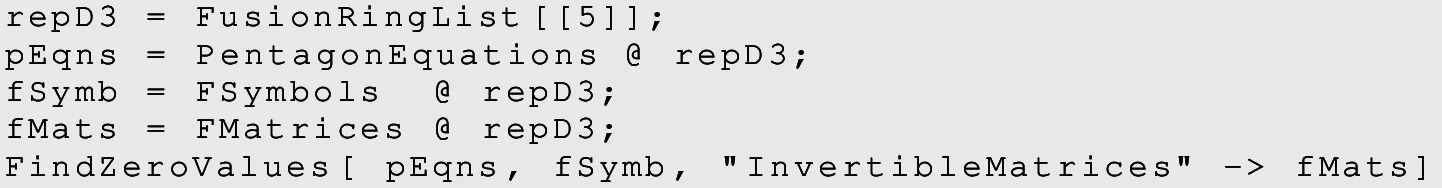

returns

**Figure:**
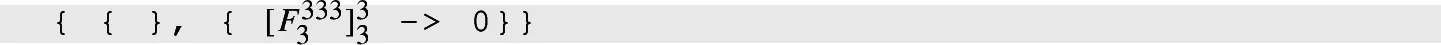

which means that there are two possible sets of zero values. Either no *F*-symbol is zero, or only . For both of these possibilities, the pentagon equations need to be solved. It turns out that the first configuration, with no zero *F*-symbols, does not lead to any solutions, and $\text {Rep}(D_3)$ is the smallest multiplicity-free fusion ring with a non-trivial zero *F*-symbol.

##### Remark 4.6

Since code listings can take up much space, LaTeX typesetting will be used to display the variables in these environments. In particular,  will be used instead of the standard output F[ a, b, c, d, e, f ] from the package. Likewise, triple dots ... will be used to condense all but a few elements of long lists whenever their content is similar to the elements that will be shown.

#### Fixing the Gauge

The pentagon equations never have a finite number of solutions because of gauge freedom. Let  be a set of numbers that denote the change of basis of the 1D vector spaces . By applying such a basis transform, the values of the *F*-symbols change as56Since the pentagon equations are derived in a basis-independent way, they are symmetric under such transforms. In particular, for every solution to the pentagon equations given by a set , the set  is also a valid solution. This allows us, even without knowing any of the values of the *F*-symbols a priori, to fix the values of certain *F*-symbols before solving the pentagon equations. In effect, we are choosing specific representatives for the orbits of the solutions under such gauge transforms. This is why we call this process fixing the gauge. Interestingly, a result of Ocneanu tells us that after fixing the gauge, the number of solutions to the pentagon (and hexagon and pivotal) equations is finite [28]. Completely fixing the gauge is often not possible, though. What we can do, however, is fix the gauge up to a discrete group of gauge transforms. After we solve the pentagon equations, we will need to check for equivalent solutions and remove these.

The definition of a fusion system already has several gauge demands in disguise. Indeed, the demand that any *F*-symbol  with a=1, or c=1 equals 1 has no effect on the categorical level. Here, we show that these demands are artificial in that it is always possible to fix a gauge for which they are true. Moreover, if such a gauge is chosen, one also has that , so any vacuum *F*-symbol, i.e., for which a=1, b=1, or c=1, must equal 1.

##### Proposition 4.7

For any set of *F*-symbols of a multiplicity-free fusion category it is possible to choose a gauge such that any vacuum *F*-symbol equals 1.

##### Proof

First, we note that any vacuum *F*-matrix must be a 1×1 invertible matrix. In particular any vacuum *F*-symbol is non-zero. Now assume a set  of values of *F*-symbols is given. We will show that there exists a set of symbols  parametrizing a gauge transform φ such that  whenever a=1, b=1, or c=1. This will be shown by listing all cases of vacuum symbols in order of decreasing number of vacuum labels and refining the gauge transform as we go along.

Denote by *E*(*a*, *b*, *c*, *d*, *e*, *f*, *g*, *h*, *i*) the pentagon equation in the gauge transformed variables:57**Case 1:** a=b=c=1

In this case also d=e=f=1 and there is only one such symbol: . This symbol is gauge-invariant, i.e.,  but from the pentagon equation58it follows immediately that .

**Case 2:** a=b=1,c≠1

In this case c=d=f,e=1 and we find that59so if we set  for all c≠1 then .

**Case 3:** a=c=1,b≠1

In this case b=d=e=f and we find that the symbol is gauge invariant. Because of the choice of φ from case 2 we find that  simplifies to  and thus  for all b≠1.

**Case 4:** b=c=1,a≠1

In this case d=e=a,f=1 and we find that60so if we set  for all a≠1 then .

**Case 5:** $$a = 1,b \ne 1,c \ne 1$$

In this case e=b,f=d and we find that61and thus its value is fixed because we already put constraints on the relevant gauge factors. By choosing suitable labels, however, we find that62or, because of our choice of φ,63and thus .

**Case 6:** $$ a \ne 1, b = 1, c \ne 1$$

In this case e=a,f=c and from64we find that .

**Case 7:** $$ a \ne 1, b \ne 1, c = 1$$

In this case we choose our labels such that the second factor in the pentagon equation equals ,65and we find that . □

In what follows, we will assume that vacuum *F*-symbols have already been set to 1, and no gauge transform is allowed to change these values.

One can apply the following procedure to fix the gauge for the other *F*-symbols. Let $\left\{ \varphi ([F^{abc}_{d} ]_{ef}) \right\}$ be a set of *F*-symbols obtained after applying a gauge transform with variables  on , i.e.66Pick an *F*-symbol whose transform has a gauge variable, say , that appears to the power 1 or -1 in Eq. (66). Set the transformed *F*-symbol equal to 1, and solve this equation for . Then, substitute this value of  into the other gauge equations and repeat this procedure with another *F*-symbol. Sometimes, this procedure can be carried through until no more free gauge factors appear in the equations. When this happens, the gauge freedom of the *F*-symbols is completely exhausted. However, we may generally run out of equations containing gauge factors with power 1 or -1 before the gauge is fully fixed. In such cases, one can continue the process using quadratic or higher-order equations in the gauge factors. Such equations do not have unique solutions for the gauge factors, which results in a partly broken gauge. The continuous symmetry is broken, but a discrete symmetry from a residual finite group of gauge transforms remains. This is not a problem when solving the pentagon equations since the number of solutions will still be finite, but afterward, duplicate equivalent solutions should be deleted.

The Anyonica package contains several functions that help deal with multiplicative symmetries, i.e., symmetry transforms that act via multiplication with specific variables. The function BreakMultiplicativeSymmetry takes a set of symmetries (that can be created using the GaugeSymmetries function) and returns a couple of (1) remaining symmetries and (2) a list of values of fixed symbols.

For example

##### Example 4.8

For the $\text {Rep}(D_3)$ fusion ring initialized in example 4.5, evaluating

**Figure:**
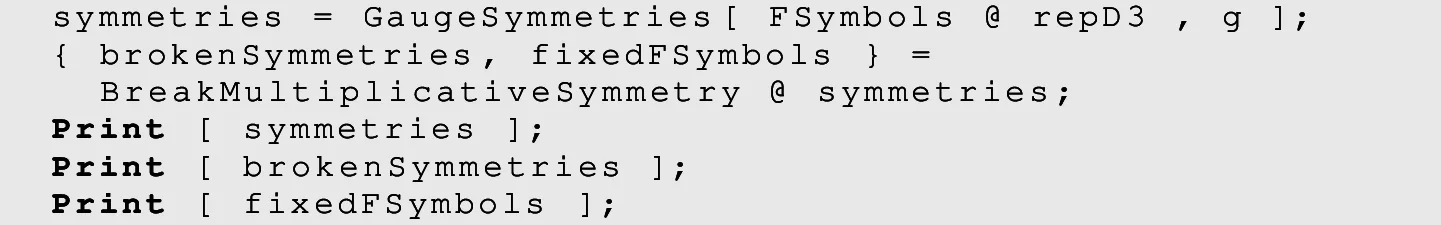

returns

**Figure:**
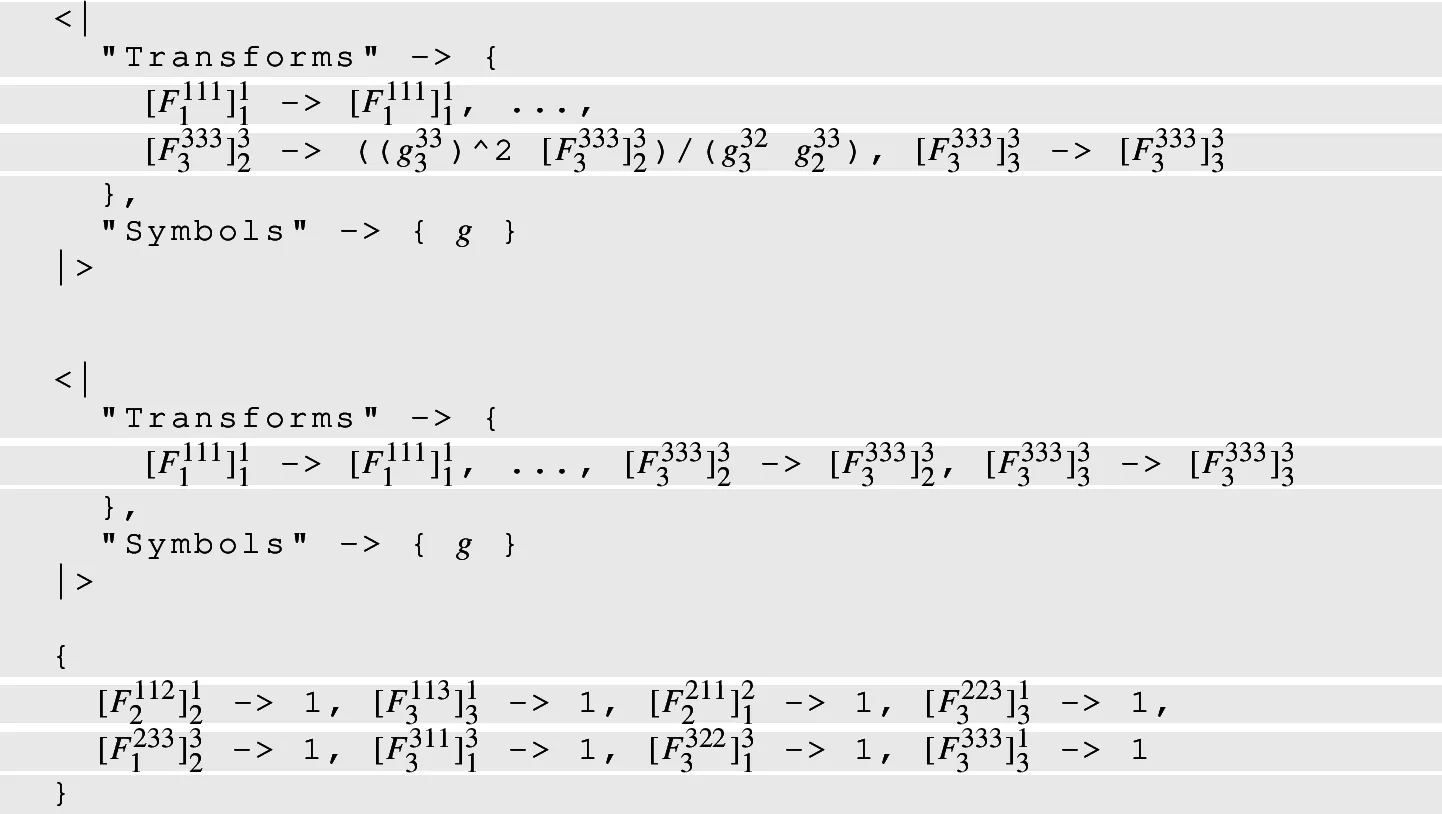

It is possible to set desired *F*-symbols to specific values by setting the option "GaugeDemands" in which case these symbols will be changed to the desired value as soon as possible in the process, to the extent that they are free to choose. Likewise, one can exclude variables from being fixed by using the "ExcludedVariables" option. While the symmetries in the example were calculated via the GaugeSymmetries function that recognizes *F*-symbols and *R*-symbols, the function BreakMultiplicat iveSymmetry accepts arbitrary symbols and transforms as long as the symmetries are multiplicative.

##### Remark 4.9

Besides the gauge symmetry, one would ideally also like to break permutation symmetry, coming from the fusion ring automorphisms. However, we did not attempt to break this symmetry while solving the consistency equations and only removed equivalent solutions afterward. One of the reasons this workflow was chosen is that it is very hard to get rid of this symmetry beforehand. The complexity of the workflow would increase a lot by adding intermediate steps to hunt down these redundancies. It might be interesting or even necessary to add this functionality in the future in order to categorify larger rings with bigger groups of automorphisms, though.

#### Solving Binomial Equations

Now that the gauge is fixed and none of the remaining variables are 0, the pentagon equations can be greatly reduced using the subset of equations with two terms. These equations have the specific form $m_{i_1} = m_{i_2}$ where the $m_{i_{j}}$ are monomials in non-zero variables. Since none of the variables can be 0, one can apply a logarithm to transform these equations to a linear system of the form $A.\vec {x} \equiv \vec {y} \mod 2\pi i \mathbb {Z}^n$, where *n* is the number of *F*-symbols. Such a system can be solved by taking a Smith decomposition of the matrix *A*.

The general form of the solution to a system of binomial equations in the variables $f_j$ is the following:

67 $$\begin{aligned} f_j = v_j \prod _{k = 1}^{\text {rk}(A)} e^{2 \pi i [Q]^k_{j} m_k} \prod _{k = 1}^{n-\text {rk}(A)} z_{k}^{[Z]^k_{j}}, \end{aligned}$$

where *v* is a constant vector, *Q* a matrix of rational numbers, *Z* a matrix of integers, the $m_k$ are integer parameters, the $z_k$ are non-zero complex parameters, and $\text {rk}(A)$ is the rank of *A*. From the RHS in (67) we see that only a finite number of lists of integers $\vec {m} = (m_1, \ldots ,m_r)$ give rise to different solutions. For each $\vec {m}$, we get a parametrization of the *F*-symbols in fewer variables than before. These parametrizations can be substituted in the remaining non-binomial equations, which become equations in the parameters $z_k$. Parametrizations for which an *F*-matrix becomes singular or for which the updated set of equations is inconsistent are removed at this point.

Typically, a new subset of binomial equations arises after the parametrization, and thus, we can repeat the process recursively until no new binomial equations are found. This procedure results in a finite number of couples consisting of (a) a system with fewer equations and fewer variables and (b) a parametrization of the *F*-symbols in these variables.

The reduction in the number of variables and equations is often quite drastic. For example, for the $\text {PSU}(2)_6$ fusion ring, all equations are solved by this procedure alone. For the $\text {HI}(\mathbb {Z}_3)$ fusion ring, we get a reduction of 41125 equations to 31 and 1212 variables to 5.

In Anyonica, the function SolveBinomialSystem solves a binomial system of equations using the Smith decomposition. It takes three arguments, equations, variables, and parameter, and returns a list of solutions parametrized by parameter. Several options can be set, such as

- "NonSingular" which is set to True if no variable is allowed to be zero,
- "ZeroValues" which is set to a list of sets of variables that are known to be 0,
- "Symmetries" which is set to a list of symmetries that should be broken,
- "InvertibleMatrices" which is set to a list of matrices of variables that should be invertible,
- "PolynomialConstraints" which can be set to a list of more general polynomial constraints (such as inequalities) that should be satisfied by the solutions,
- "UseDatabaseOfSmithDecompositions" which can be set to True in order to reuse previously calculated Smith decompositions, and
- "StoreDecompositions" which can be set to False in order not to waste time storing decompositions.

##### Example 4.10

After substituting  and fixing the gauge we can solve the subsystem of binomial equations using the SolveBinomialSystem function.

**Figure:**
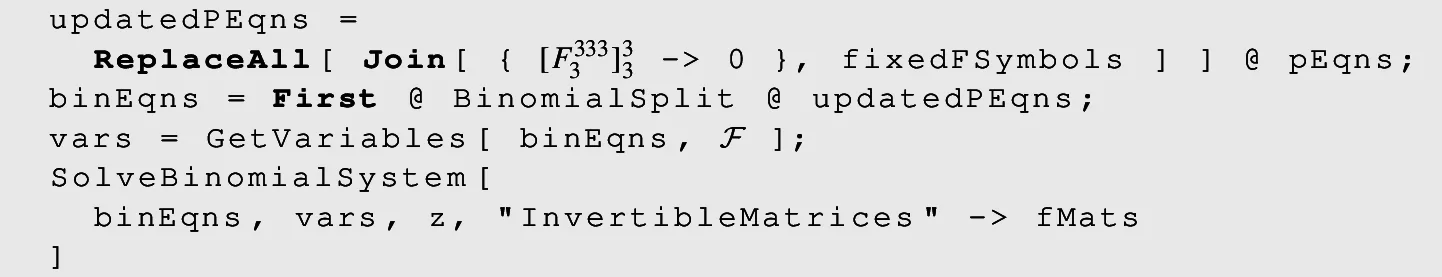

then we obtain 6 parametrizations of the *F*-symbols in terms of one parameter $z_1$

**Figure:**
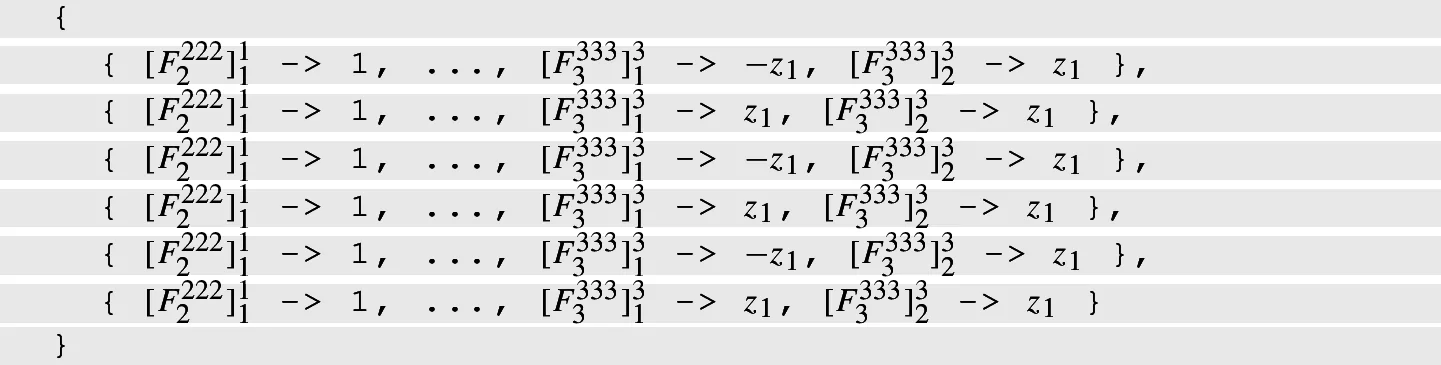

By substituting these parametrizations into the 10 remaining equations, we obtain 6 new, greatly simplified systems of equations.

To repeatedly reduce a system of equations with more than two terms, the function ReduceByBinomials can be used. It takes four arguments, sumEquations, twoTermEquations, variables, and parameter. Besides all the options it inherits from SolveBinomialSystem, it also takes the option "SimplifyInterme diateResultsBy" (with default Identity), which can be set to a function that simplifies intermediate solutions before substituting them. This is primarily useful if the polynomial equations contain non-integer coefficients. While this situation can occur when solving the pentagon equations, the non-integer coefficients are always cyclotomic integers at this point in the calculation. Mathematica typically simplifies combinations of these coefficients by default. However, the coefficients that appear in the hexagon equations are solutions to the pentagon equations and can be pretty wild. Since the solutions to the hexagon equations are always phases, it is often desirable to express intermediate coefficients as phases.

#### Reduction via Linear Polynomials

When no more binomial equations are present we can reduce the system of equations even further by using equations that are linear in a certain variable, i.e., of the form

68 $$\begin{aligned} z p + q = 0, \end{aligned}$$

where *p* and *q* are polynomials that are independent of the variable *z*. From this equation it is clear that either

69 $$\begin{aligned} z = - q / p,\quad \text {or}\quad p = 0. \end{aligned}$$

The process of reduction is the following. Let $D = (l_P, l_A, l_Z)$ be a triple of lists, to which we will refer to as ‘the data’, with the following purpose:

- $l_P$ is the list of polynomials that we want to reduce. During the reduction process the polynomials in this list will be updated, and polynomials equal to 0 will be deleted. Initially $l_P$ contains the list of all polynomials we want to reduce.
- $l_A$ is the list of assumptions that are made during the reduction process. Here polynomials will be stored that are assumed to be non zero. $l_A$ is initially empty.
- $l_Z$ is the list of deduced values of variables. During the reduction process, more values will be added and previous values will be updated. $l_Z$ is initially empty.

If at any point during the reduction, $l_P$ contains a non-zero number, $l_A$ contains 0, or any of the values in $l_Z$ becomes zero then the data at that point is regarded as invalid. The following procedure is then applied to reduce the list of polynomials.

Let $L_{\textrm{reduced}}$ be an empty stack.

1. Find the *simplest* linear polynomial in $l_P$.
2. If there is no linear polynomial, throw *D* on $L_{\textrm{reduced}}$. If there is, say zp+q, then
  - If *p* is monomial, solve for *z* and update *D* to $D_{\textrm{new}}$ by substituting the value of *z* in *D*, and adding its value to $l_Z$. If $D_{\textrm{new}}$ is valid then remove all 0 polynomials from it, convert all rational functions to polynomials, and repeat step 1 with the $D_{\textrm{new}}$ as input.
  - If *p* is not monomial then create two copies of *D*, say $D_1$ and $D_2$, and
    (i) solve for *z* under the assumption that p≠0. Update $D_1$ to $D_{\textrm{new},1}$ by substituting the value of *z* in $D_1$, adding *z* to $l_{P_1}$, and adding *p* to $l_{A_1}$. If $D_{\textrm{new},1}$ is valid, remove all 0 polynomials from it, convert all rational functions to polynomials, and repeat step 1 with $D_{\textrm{new},1}$.
    (ii) Update $D_2$ to $D_{\textrm{new},2}$ by reducing all polynomials in $D_2$ (including zp+q) modulo *p*. If $D_{\textrm{new},2}$ is valid, remove all 0 polynomials from it and repeat step 1 with the updated data.

In each step where a linear polynomial is encountered the list of polynomials gets either reduced by at least 1 element or, in the case of an invalid system, the process quits. The algorithm, therefore, always stops in a finite time.

After performing these steps one should have a stack of triples

$$ \{ ( l_{P;1}, l_{A;1}, l_{Z;1} ), \ldots , ( l_{P;n}, l_{A;n}, l_{Z;n} ) \}, $$

all corresponding to reduced systems of equations. Note that while the systems of equations have fewer equations and variables, the size (in bits) can become large. The systems created this way are, however, easier to tackle using Gröbner bases algorithms combined with some tricks.

The function ReduceByLinearity performs the reduction of a system of polynomial equations by linear equations. It takes two standard arguments polynomialList and x, where the variables of the polynomials should be of the form x[1],...,x[n]. The *simplest* linear equation is based on (1) the lexicographic weight of the function { MonomialQ[p], MonomialQ[q] } which prefers fractions of monomials but prioritizes fractions where only *p* is monomial over those where only *q* is monomial, and (2) LeafCount[p/q] which provides a simple measure of the complexity of an expression. With the option "LinearReductionWeight", a custom weight function can be given to decide the simplest polynomial. This can be necessary since the execution time, maximum memory used, the size, and the form of the result depend greatly on the order of the variables that are solved for. Extra options can also be set to parallelize evaluation and control the memory pressure.

##### Remark 4.11

- This procedure can also be used to reduce the system of binomial equations to a great extent (which is what J.K. Slingerland’s code, see Sect. A does). I still decided to use F. Verstraete’s idea to use the Smith decomposition since these decompositions can be stored. This allows one to check and redo calculations much faster. The Smith decomposition does have some serious drawbacks, however. Firstly, the required memory for the algorithm scales quite badly with the size of the system. Secondly, the Smith decomposition returns all solutions to the binomial system in one go. For example, for the $\text {SO}(7)_2$ fusion ring, the outcome of ReduceByBinomials is a list of 64 reduced systems. Many of these intermediate solutions do not result in an actual solution and are thus a waste of space and time. Lastly, if a crash occurs during the computation of the Smith decomposition, it is very hard to recover the computations performed so far. While the linear reduction can also suffer from memory blowup, this can often be prevented by choosing a different order in which the variables are substituted. Because it is easy to keep track of the steps of the linear reduction, it is easier to avoid intermediate memory blowups. The linear reduction also avoids the blowup in the number of solutions since quadratic and higher order terms are not substituted for. For bigger systems of pentagon equations, it might be more beneficial to use the linear reduction directly after fixing the gauge instead of reducing the system via the Smith decomposition.
- While $l_A$ is initialized as an empty list when solving all systems, it might be beneficial to initialize it to a list of determinants of the *F*-matrices. This does not affect the eventual solutions found since, in the last step, all singular solutions are removed. It might affect computation speed, however.

#### Incremental and Parallel Gröbner Bases

The penultimate step in solving the pentagon and hexagon equations is the computation of Gröbner bases. A Gröbner basis of a set of polynomials is a set of polynomials that has the same roots as the original system, but from which it is easier to extract certain properties of the system. Both the form of the basis and the properties that can be extracted depend on a given monomial ordering. A Gröbner basis in “Lexicographic order” is especially interesting for our purpose. It has a polynomial upper triangular form: the first polynomial contains only one variable, the second contains only two variables of which one is shared with the first polynomial, and so on. While the number of variables at this stage is quite low, the number and size of equations could still be high. Typically, though, a lot of the equations are redundant. Therefore, we implemented the function IncrementalGroebnerBasis, which creates such a basis incrementally. It takes two arguments, polynomials and variables, and works as follows. Let $l_P$ be the list of polynomials given by polynomials.

1. First find several of the *simplest* polynomials and compute a Gröbner basis, say $\mathcal {G} = \{p_1,\ldots , p_n\}$, for these.
2. The system $l_P$ is then reduced via $\mathcal {G}$ as follows. Each polynomial *q* in $l_P$ can be written as a linear combination $q = a_{q;1} p_1 + \cdots a_{q;n} p_n + r_q$ where $a_{q;1}, \ldots , a_{q;n}$, and $r_q$ are polynomials such that $r_q$ is indivisible by any of the $p_i$, i.e., is the remainder of the division of *q* by $\{p_1,\ldots ,p_n \}$. Since the $p_i$ are assumed to be zero, one can replace all polynomials *q* by $r_q$.
3. Next all 0 polynomials and all duplicate polynomials are removed from $l_P$. If no polynomials remain then $\mathcal {G}$ is a Gröbner basis for the system. If some polynomials remain then we add $\mathcal {G}$ to the reduced $l_P$ and repeat steps 1, 2, 3 with the updated $l_P$.

The Gröbner bases are calculated using the standard GroebnerBasis function that comes with Mathematica.

For the pentagon and hexagon equations we tackled, such a basis typically contains less than 20 polynomials and less than 5 variables.

Just like ReduceByLinearity, the memory consumption of IncrementalGroebnerBasis can easily blow up. To reduce the risk of memory overflows, we made it possible to set certain options by hand. The option "Cutoff" can be set to a number between 0 and 1, determining which fraction of all polynomials to consider in step 1. The option "GroebnerWeightFunction" determines how *simple* a polynomial is, and by default, it is the function PolynomialDegree. In some cases, it might be beneficial to choose another measure of complexity, such as e.g. the number of terms or a measure based on the complexity of the coefficients. If the polynomial system contains symbolic roots of polynomials as coefficients, it might be interesting to set the option "ReduceRoots" to True. This implies that the algorithm will reduce algebraic expressions containing roots to roots of single polynomials after each reduction step. Without intermediate simplification of this kind, large algebraic expressions without variables often linger in the system while they should either be 0 or, if not, allow us to conclude that the system has no roots. A custom function to perform simplifications can also be given by setting the "SimplifyIntermediateResultsBy" option.

The order of the variables given to the GroebnerBasis function also plays an important role. Different permutations of the list of variables given to the function can result in considerable differences in execution time, memory consumption, and output complexity. The function ParallelGroebnerBasis was constructed to deal with this issue. For every available parallel kernel, the function chooses a permutation of the variables and starts the task of computing a Gröbner basis. The moment one of the tasks finishes, it returns the Gröbner basis, and all other tasks are killed. This way, one can obtain speedups of factors 10000 or higher using only a few extra cores.

##### Remark 4.12

For some systems, it is not beneficial to compute Gröbner bases in the first place. If the results from the linear reduction are systems in one variable, it is often much faster to find the lowest degree polynomial in that variable, solve it, and remove solutions that do not satisfy the assumptions from $l_A$ or are incompatible with the other equations in $l_P$. It might even be beneficial to do this for systems with two or more variables (which might or might not have equations containing only one variable), but at the moment, only the check for systems with one variable is implemented.

#### Solving the Pentagon Equations

To find roots of Gröbner bases, the built-in function Reduce was used. This function returns a proposition that provides the solutions for a polynomial system with a finite number of solutions. Sometimes, a closed form cannot be found or is too complicated, so Mathematica solves the system in terms of Root expressions. These are symbolic placeholders, in our case of the form Root[*p*(*x*), *n*] where *p* is a polynomial and *n* denotes the index of the root according to a specific ordering. Such an expression contains all the information to specify the root it represents exactly. One can, in particular, obtain the numeric value of a Root expression to arbitrary precision and combine and simplify root objects symbolically.

All the functionality described so far is bundled in the function SolvePentagon Equations. By default, SolvePentagonEquations takes only a single argument: a fusion ring. There are, however, a large number of options one can set. Each option for the functions discussed so far is also valid for SolvePentagonEquations. Other options come from built-in functions such as GroebnerBasis, Reduce, and there are also some other options which can looked up in the documentation of the package. For multiplicity-free fusion rings up to rank 6, only several of the options are necessary. To categorify a generic fusion ring of higher rank, however, one likely needs to experiment with various options to avoid bottlenecks and crashes. It is even quite likely that more fine-tuned control is required and that, for example, it is necessary to swap the order of specific steps, substitute solutions to subsystems, or manually find and solve certain polynomial equations. Various extra functions were implemented to accommodate this need. These include functions that manipulate polynomial expressions, gauge symmetries, fusion rings, fusion categories, and more. Anyonica contains more than 170 different documented functions at the moment of writing. By evaluating  in Mathematica, one gets a list of all functions with clickable hyperlinks that reveal usage information.

##### Example 4.13

The result from evaluating the following code

**Figure:**
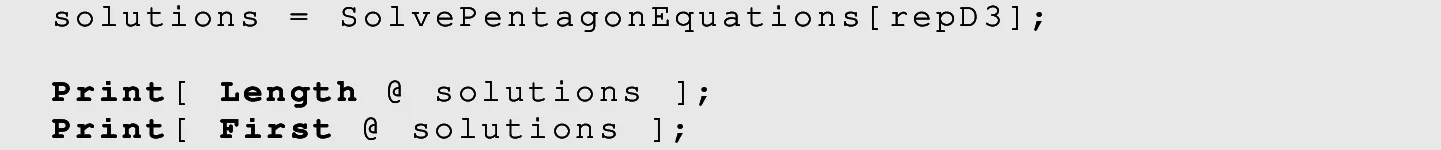

is

**Figure:**
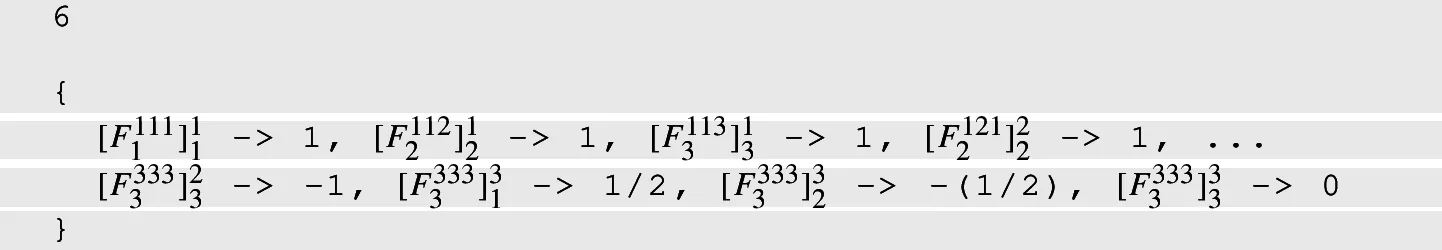

#### Removing Equivalent Solutions

Two solutions to the pentagon equations are equivalent (or, fully equivalent) if and only if they can be transformed into each other via a combination of

- a gauge transform:  for some set of numbers  and
- a fusion ring automorphism:  where $\sigma \in S_r$ is a permutation for which  for all $a,b,c \in \textbf{L}$.

After the pentagon equations are solved, there are typically several equivalent solutions, and it is desirable to remove such redundant solutions. Finding out whether two fusion systems, say $\Phi _1,\Phi _2$ respectively with *F*-symbols $\left\{ [ \overset{_\textbf{1}}{F}\phantom {F}^{a,b,c}_d ]^e_f \right\}$ and $\left\{ [ \overset{_\textbf{2}}{F}\phantom {F}^{a,b,c}_d ]^e_f \right\}$ are gauge-equivalent is equivalent to checking whether the system of equations70has any solutions for the non-zero variables . In [77] an algorithm, based on the Hermite normal form, is presented to check this.

To check whether two fusion systems, $\Phi _1$ and $\Phi _2$ are fully equivalent one could permute the labels of the *F*-symbols of $\Phi _1$ and check equivalence with the *F*-symbols of $\Phi _2$ for every permutation. When applying this algorithm to a large list of fusion systems, many computations only depend on the fusion ring and the configuration of *F*-symbols that are 0. These computations are often repeated, and this approach becomes unfeasible for fusion rings of high rank with many solutions to the pentagon equations. This observation led us to use an alternative approach. Instead of checking whether two fusion systems are equivalent by checking whether the system (70) has a solution, we will create an invariant that is strong enough to decide whether any two fusion systems with the same fusion ring are equivalent. Before explaining how to construct such an invariant, we will introduce some useful definitions.

First, we need to distinguish between expressions of *F*-symbols that are formally gauge-invariant and those that are practically gauge-invariant.

##### Definition 4.14

Let $\Phi = (\textbf{L},*,\textbf{N},\textbf{F})$ be a multiplicity-free fusion system and $\mathcal {L}_\Phi$ be the ring of rational functions in formal *F*-symbols of Φ. Its elements, the formal *F*-symbols, are regarded as functions from $\textbf{L}^6 \rightarrow \mathbb {C}$ without assigned values or constraints, and the multiplication and addition in $\mathcal {L}_\Phi$ are the usual multiplication and addition of functions.

- An element $\rho \in \mathcal {L}_\Phi$ is called **de jure gauge-invariant** if it is invariant under any gauge transform φ, i.e. $\varphi (\rho ) = \rho$ for all φ.
- Let $\rho \in \mathcal {L}_\Phi$ and let $[\rho ]_{\Phi }$ be ρ where its *F*-symbols have been replaced by their values from Φ. ρ is called **de facto gauge-invariant** if $[\rho ]_\Phi$ is well defined and $[\varphi (\rho )]_\Phi = [\rho ]_\Phi$ for all gauge-transforms φ.

##### Example 4.15

For the multiplicity-free category $[\text {Adj}(\text {SO}(16))]_{1,1,1}$ the *F*-symbol  is not de jure gauge-invariant since , but it is de facto gauge-invariant since its value is 0 and therefore unaffected by any gauge transform.

Note that it is not necessary to know the values of the *F*-symbols to determine whether an element ρ of $\mathcal {L}_\Phi$ is de jure gauge-invariant. To determine whether ρ is de facto gauge-invariant, however, certain extra information about the values of the *F*-symbols is required. Which information depends on the form of ρ. If, for example, ρ is a rational monomial in formal *F*-symbols, we only need to know which *F*-symbols are 0.

The invariant is based on the construction of a *gauge-split basis* of a fusion system.

##### Definition 4.16

Let $\Phi = (\textbf{L},*,\textbf{N},\textbf{F})$ be a multiplicity-free fusion system and $\mathcal {M}_\Phi \subset \mathcal {L}_\Phi$ be the subset of rational monomials (RMs) in formal *F*-symbols. A **gauge split basis** of Φ is a tuple (*I*, *D*) where $I = (\iota _1,\ldots ,\iota _m) \in \mathcal {M}^m_\Phi$, $D=(\delta _1,\ldots ,\delta _n)\in \mathcal {M}^n_\Phi$ and for which the following properties hold.

1. For each RM ρ in *I* and *D*, $[\rho ]_\Phi$ is well-defined.
2. Each element of *I* is de facto gauge-invariant.
3. Each element of *D* is gauge-dependent and moreover the values of each of the $\delta _i$ can be independently set to any non-zero value by performing a suitable gauge transform.
4. For any RM $\rho \in \mathcal {M}_\Phi$ there exist m+n unique integers $a_1,\ldots ,a_m, b_1,\ldots , b_n$ such that
  71 $$\begin{aligned} \rho = \prod _{i = 1}^m \iota _i^{a_i} \prod _{i = 1}^n \delta _i^{b_i} \end{aligned}$$

We then have the following proposition

##### Proposition 4.17

For every multiplicity-free fusion system, there exists an infinite number of gauge-split bases.

##### Proof

See section 4 of [78]. □

Several functions are implemented in Anyonica to play around with gauge invariants.

- GaugeSplitTransform takes a fusion ring and returns a couple of (1) a matrix *M* and (2) a number *m* such that $\prod _{j}\left( [L_F]^j \right) ^{[M]^j_i}$ equals $\iota _i$ for i≤m and $\delta _{i-m}$ for m<i. Of those elements the ones whose values evaluate to 0 appear first. Here $L_F$ is the list of *F*-symbols that gets returned by the FSymbols function and can be in any order.
- GaugeSplitBasis takes a fusion ring and returns a gauge-split basis as a couple of lists. The first list corresponds to the gauge-invariant RMs and the second to the remaining gauge-dependent RMs.
- GaugeInvariants takes a fusion ring and returns only the first list from GaugeSplitBasis.

For all these functions, one can set option "Zeros" to a list of symbols that are 0. By default, these functions also include *R*-symbols and pivotal symbols in the gauge invariants (which has no meaning if the categories are non-braided), but by setting the option "IncludeOnly" to {"FSymbols"}, it is possible to return invariants only in *F*-symbols. These functions then form the core of the function GaugeEquivalentQ, which takes a fusion ring and two sets of *F*-symbols and returns true if the solutions are gauge equivalent.

##### Example 4.18

The following code

**Figure:**
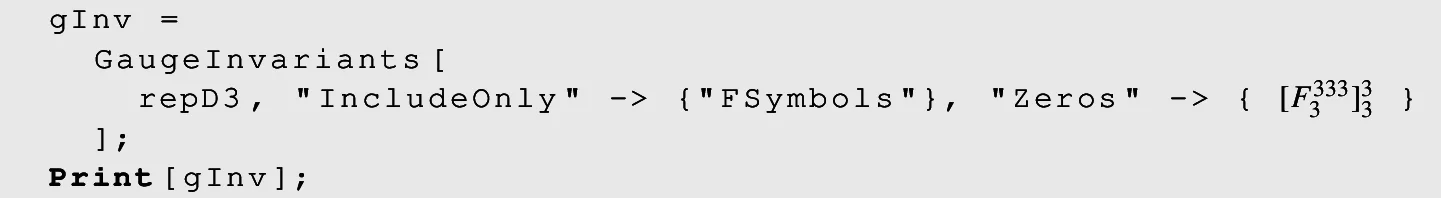

returns

**Figure:**
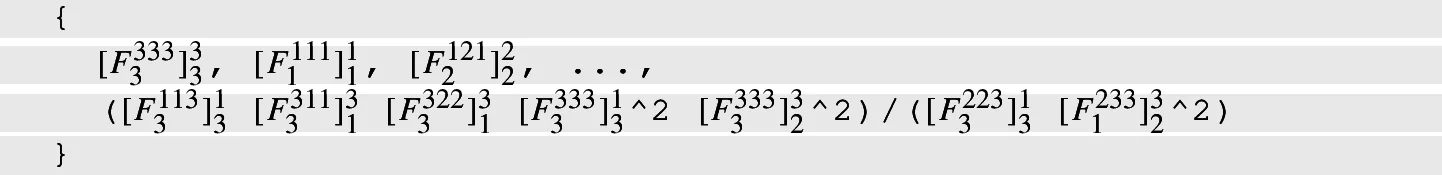

Using these invariants one can easily see which solutions are gauge equivalent. The following code

**Figure:**
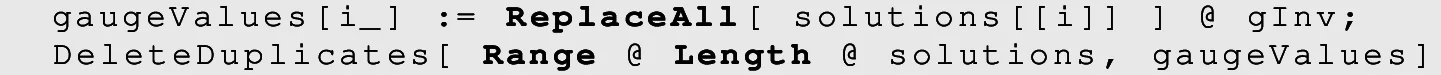

returns the indices of the unique solutions, in this case

**Figure:**
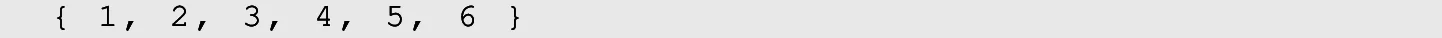

Since we started with six solutions, we find that none of the solutions are gauge equivalent. We will see, however, that only three unique solutions are left when we delete solutions based on full equivalence.

To check whether two solutions are equivalent up to a gauge transform in combination with a fusion ring automorphism it is possible to create a complete invariant of a fusion system as follows. Let Φ be a fusion system with fusion ring $\mathcal {R}$. Let $S = \textrm{Aut}(\mathcal {R})$ be the group of fusion ring automorphisms of $\mathcal {R}$, and (*I*, *D*) a gauge split basis for Φ. Let $[S(I)]_\Phi$ be *S*(*I*) where each *F*-symbol has been replaced by its value from Φ, then, by construction, $(S(I),[S(I)]_\Phi )$ is a complete invariant of the fusion system Φ. Since the construction of *S*(*I*) only depends on the configuration of *F*-symbols that are 0, and only three multiplicity-free fusion systems up to rank 7 have different configurations of *F*-symbols that are 0, it is much more efficient to construct *S*(*I*) first and then evaluate it for the various fusion systems.

In Anyonica the function SymmetryEquivalentQ can be used to check for full equivalence of two solutions to the pentagon equations. For deleting multiple solutions one can use the more efficient DeleteEquivalentSolutions. Since both SymmetryEquivalentQ and DeleteEquivalentSolutions check for equality to remove solutions, they are by default conservative: they might decide two fusion systems are inequivalent because equality between symbolic expressions is not trivial.

##### Example 4.19

Any computer algebra system has to deal with how much it should automatically simplify symbolic expressions. Mathematica is quite conservative, and for all but the most trivial cases, the user must choose when to simplify. The following, almost trivial, equality is, for example, not simplified by default

**Figure:**
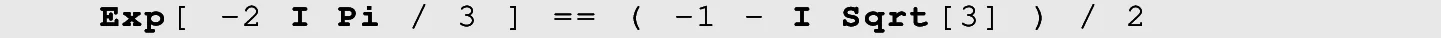

However, applying a function such as RootReduce transforms this equality to True.

To avoid this situation, it is possible to set the option "PreEqualCheck" to a function of choice that will be evaluated on any two arguments before they are checked for equality. Note that, for example, if numeric simplification with low accuracy is used, it becomes possible that too many solutions are removed. To avoid this from happening, built-in functions such as N (which gives a numerical approximation) and FullSimplify (which aggressively (over)simplifies expressions) should be avoided. Rather, safe variants such as RootReduce should be used. It is also possible to delete redundant solutions using numerical methods, which are almost always faster. By setting the option "Numeric" to True each gauge invariant is calculated with an accuracy of (by default) 64 decimal digits and infinite precision (which means that Mathematica uses as many extra digits required in intermediate calculations to ensure that those 64 decimal digits are correct up to rounding of the last digit). A different accuracy can be set if desired using the "Accuracy" option.

##### Example 4.20

The following code

**Figure:**
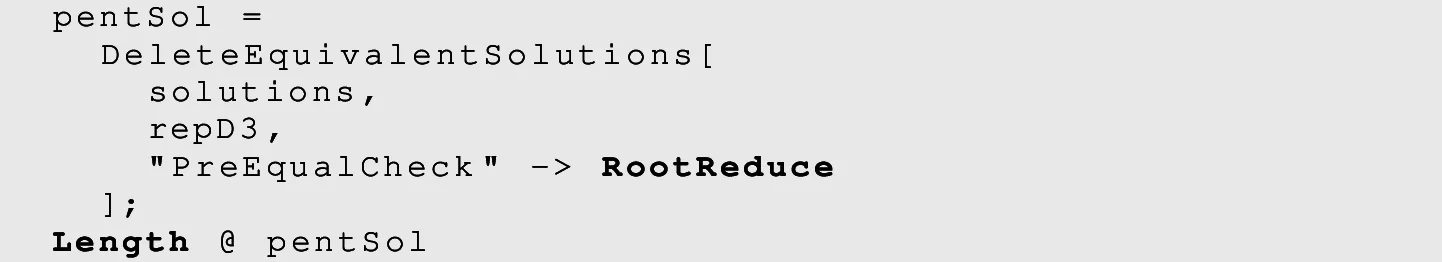

returns

**Figure:**
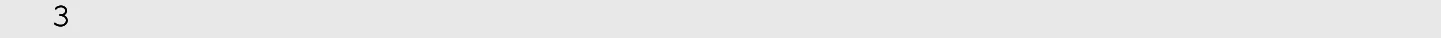

which means that checking for gauge invariance alone is not enough to remove redundant solutions as some gauge inequivalent solutions are related by automorphisms.

We removed redundant solutions using both the symbolic and numeric methods for each set of solutions to the pentagon equations and checked whether these agreed. This was always the case for an accuracy of 1000 decimal digits. While the symbolic method should, in principle, always give a correct result, it is still important to check the results by other means, whenever possible.

In [27] tables of small invariants, based on *S*(*I*), are provided that show that all fusion systems provided by Anyonica and the anyonwiki are, indeed, inequivalent.

#### Fixing a unitary gauge

The specific values of the *F*-symbols are gauge-dependent, and thus, we can manipulate the gauge to put solutions to the pentagon equations, as provided by the SolvePentagonEquations, in a more appealing form. A particularly interesting gauge is one for which all *F*-matrices are unitary. Not all solutions permit such a unitary gauge, though, but if they do, we would like to represent them in such a gauge.

If a set of *F*-symbols is in a unitary gauge then we know that72for all admissible *F*-symbols, and vice versa. Since the left hand side of this equation is gauge-invariant we already know its value, even for a set of non-unitary *F*-symbols.

Now assume that we have a set of *F*-symbols that is not in a unitary gauge. Finding a unitary gauge then comes down to finding a set of gauge variables  such that73where  is the inverse matrix of . Since we know all the values of the *F*-symbols involved, this is a set of equations in the variables . These equations can be tackled one by one in a similar manner that we fixed the gauge before solving the pentagon equations: we pick the simplest equation, solve it, substitute the solution into the rest of the equations, and continue. At some point, equations might appear that only contain higher powers of the variables $$h^{a,b}_c$$ but, as was the case with fixing the gauge, we are free to choose any solution for the $$h^{a,b}_c$$ variable in consideration. The difference between the various solutions for the variable $$h^{a,b}_c$$ is that certain *F*-symbols will differ by a phase factor when comparing solutions. However, if we look at (73) we see that the right-hand side does not care about phases, and the left-hand side does not care about gauge transforms. Therefore, this redundancy is harmless, and we can use the first solution for the $$h^{a,b}_c$$ that the Solve function returns. It is always possible to solve for all $$ \left\{ h^{a,b}_{c}\right\} $$, but this only implies that the specific equations we used to find such a solution are satisfied. The final step in the algorithm consists of checking whether all equations of the form (73) are satisfied. If so then we can apply a gauge transform with gauge variables  to the original set of *F*-symbols  to put it into a unitary gauge. Note that also here there is a redundancy of the phases of the various , but this does not matter for the same reason that the redundancy in the phases for the various $$h^{a,b}_{c} $$ do not matter.

### Solving Hexagon Equations

The hexagon equations (26) and (27) have a structure that is very similar to the pentagon equations. In particular, per solution to the pentagon equations, there are, up to gauge equivalence, only a finite number of solutions to the hexagon equations (see remark 2.33 of [28]). The same tools we used to solve the pentagon equations were also used to solve the hexagon equations. There are a few important differences, however.

Since we solve the hexagon equations per solution to the pentagon equations, the only variables that appear are 1-dimensional invertible *R*-matrices. The *R*-symbols are, in particular, never 0.

To solve the pentagon equations, we already fixed the gauge up to a certain degree for the *F*-symbols, and therefore, we can now only fix the remaining degrees of freedom that leave the *F*-symbols invariant. To do so, the function RestrictMultiplicativeSymmetries was implemented. It takes a list of symmetry transforms symmetries, a set of variables variables, and a symbol g that denote the gauge variables $\{$g[a,b,c]} as arguments and returns a set of gauge transforms that leave the set variables invariant. The remaining gauge can then be fixed the same way as for the *F*-symbols. For most fusion rings, once the gauge for the *F*-symbols was fixed, no gauge freedom was left for the values of the *R*-symbols. Thus, one should not expect a significant reduction in variables.

Once the gauge is fixed we can apply the usual functions such as ReduceBy Binomials, ReduceByLinearity, and IncrementalGroebnerBasis. All this functionality is implemented in the function SolveHexagonEquations, which takes one argument, the fusion ring, and several options. The most important option is "Knowns", which can be set to a solution to the pentagon equations. Without this option, SolveHexagonEquations would assume that the *F*-symbols are variables and try to find their values as well. The other important options overlap with those of SolvePentagonEquations.

#### Example 4.21

The following code

**Figure:**
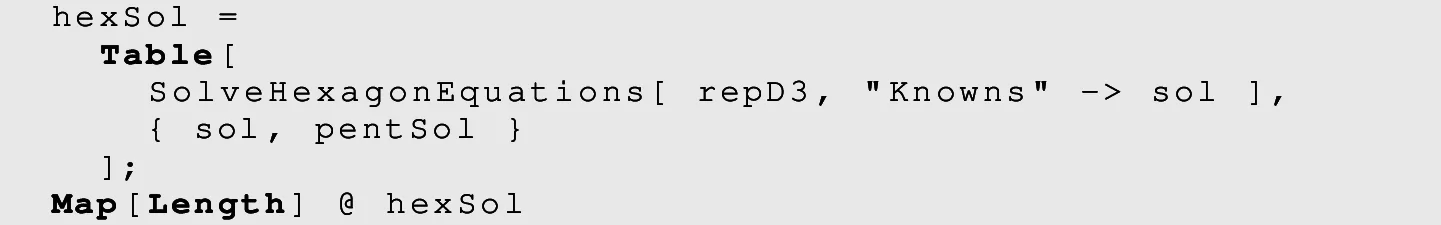

returns

**Figure:**
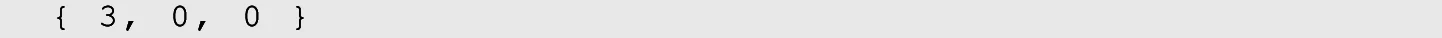

which means that the first solution to the pentagon equations has at most three compatible braided structures, and the other ones have none. It turns out there are actually exactly three braided structures since the following code

**Figure:**
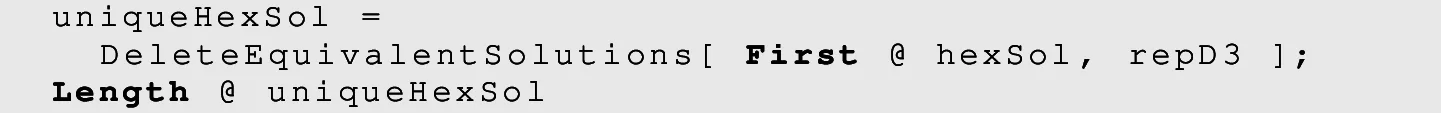

returns

**Figure:**
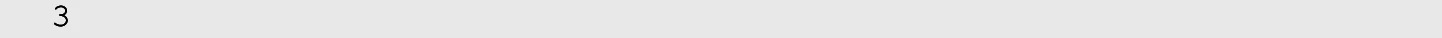

There is one catch, however. While the function SolveBinomialSystem (that forms the core of ReduceByBinomials) works quite fine for pentagon equations, it does not perform nearly as well for hexagon equations. The cause of this problem lies with the Smith decomposition. On the one hand, the solutions to the pentagon equations often contain root expressions, i.e., expressions containing *n*th roots or even expressions containing formal roots of the form Root[*p*(*x*), *n*]. On the other hand, the matrices that result from taking a Smith decomposition often contain big integers. Eventually, these integers are the exponents with which the root expressions appear in the solution to a binomial system. If the root expressions were just integers, then Mathematica has no issues canceling fractions of gigantic integers. Fractions of root objects, raised to huge powers, form a big problem, however. Moreover, even if the binomial system can be solved, it often contains many solutions. In contrast to the pentagon equations, the main restriction to the number of solutions of the binomial equations comes from the equations containing more than two terms. Eventually, most of the solutions to the binomial equations fail to satisfy the other equations, but they do cause a blowup of required memory and time.

For some fusion rings, solving the hexagon equations by first reducing the system via binomial equations was impossible. In these cases, it was, however, quite easy to solve the equations via the ReduceByLinearity function in combination with the option "SimplifyIntermediateResultsBy" to simplify intermediate results as soon and frequently as possible.

After solutions for the hexagon equations have been obtained, we need to remove the equivalent solutions again since some discrete gauge freedom might remain for the *R*-symbols. This is done the same way as for the *F*-symbols, but now we use a gauge-split basis that includes *R*-symbols to distinguish solutions.

### Solving Pivotal Equations

To find pivotal structures for a given (braided) fusion system $\mathcal {C}$, we must solve the pivotal equations (25). While Anyonica can solve these equations, they are so simple that Mathematica’s built-in Solve function is much more efficient. As always, once a set of solutions has been found, we must eliminate redundant solutions.

Since the values of the left quantum dimensions  are gauge invariants, and different for every solution, only fusion ring automorphisms can cause redundancy in the solutions. These automorphisms must be such that, up to gauge transforms, they have no effect on the *F*-and *R*-symbols and map the quantum dimensions of one solution to the quantum dimensions of another. Once the redundant solutions are removed, the pivotal structures can be added to the data of the (braided) fusion system $\mathcal {C}$.

In order to ease calculations with a graphical language, we applied a gauge transform such that every pivotal coefficient of a non-self-dual particle equals 1. This implies that in the list of stored fusion categories in Anyonica, there are pivotal fusion systems that correspond to the same solution to the pentagon equations but have slightly different *F*-symbols.

### Correctness of the Calculations

Despite being open-source, Anyonica relies on Mathematica which is proprietary software. This makes it impossible to formally prove correctness of the results, and one should be cautious with describing the obtained results as a classification. As pointed out in the introduction, for the census to be a classification, three main properties must be satisfied.

1. **Correctness.** Each fusion system corresponds to a category. In other words, each set of skeletal data solves the pentagon, hexagon, and pivotal equations.
2. **Completeness.** The census contains all categories. In other words, the census contains the skeletal data of all multiplicity-free categories up to rank 7 as well as all compatible braided and pivotal structures.
3. **Uniqueness.** None of the pivotal (braided) fusion systems are equivalent.

At the moment of writing, the authors of this paper know of no formal proof that any of the three properties holds. It is, however, possible to provide evidence for each property.

Since the data provided by Anyonica is symbolic, the first property can be checked using symbolic algebra. In particular, the correctness of the data has been verified by using the functions

- RootReduce, which expresses algebraic combinations of roots of polynomials as a root of a single polynomial, and
- N[#,{Infinity,1000}] &, which calculates the exact numerical value of its argument with 1000 correct decimal digits and uses an arbitrarily big extra internal precision to guarantee correctness of those digits (up to rounding of the last digit).

The data has also been independently verified by the TensorCategories.jl package[79] with symbolic algebra provided by the Oscar package[80]. This package is developed independently of Anyonica and uses different methods to verify the correctness of the data. Therefore, we think there is good evidence the data is correct.

In [27], the first author provides tables of small invariants that allow one to identify a multiplicity-free pivotal (braided) fusion category with rank 7 or less. In particular, it follows from the provided tables, that under the assumption that the provided fusion systems are correct, they must all be inequivalent. So the first property implies the third one.

The second property is by far the hardest one to provide evidence for. In contrast to verifying the correctness of the data, which can be done using different software packages and algorithms, it is much harder to verify the correctness of a complete package. Therefore, to reduce the risk of critical bugs and enhance the transparency of calculations, a logging system was implemented in Anyonica. For each multiplicity-free fusion ring up to rank 7 there are log files that explain the critical computations and at which point intermediate solutions were rejected and the reasons why. The most time-intensive steps, consisting of matrix decompositions and substitutions of well-chosen variables, and their results have been logged. These can, in principle, be checked using other languages as well.^3^ Therefore, the log files, at least theoretically, provide a way to check the completeness of the set of fusion categories belonging to a single Gröthendieck ring. It is, however, unfeasible to check all log files since there are too many.

While this paper was under review, it was discovered that the original data from Anyonica was incomplete. The zesting procedure, applied to $\text {SU}(6)_2$ categories, predicts [36, 81] the existence of fusion categories with $\text {FR}^{7,1,2}_{11}$ fusion rules. These categories were, however, not found using Anyonica due to a subtle bug in the code that uses symmetries to find zero values. After fixing this bug, the predicted categories were obtained. Therefore, one should remain careful with regarding the census as a classification.

## Results

By using Anyonica, the pentagon, hexagon, and pivotal equations for all but two multiplicity-free fusion rings up to rank 7 were solved, and all redundant solutions were removed. For the two exceptions, namely $\text {PSU}(2)_{12}$ and $\text {PSU}(2)_{13}$, the systems of pentagon equations were too big to handle. The categorifications of $\text {PSU}(2)_{2n+1}, n \in \mathbb {N}$ are interestingly already classified [46], and the *F*-and *R*-symbols can be easily calculated by the alatc package from Ardonne (see A for more info). For $\text {PSU}(2)_{2n}, n \in \mathbb {N}$ only the pivotal categorifications are classified (see appendix of [82]). While the same paper argues that it is most likely the case that all fusion categories are pivotal and therefore most likely all fusion categories with $\text {PSU}(2)_k,k\in \mathbb {N}$ are classified, we will not make such an assumption. We therefore propose the slightly weaker result of a census of all pivotal multiplicity-free fusion categories up to rank 7 which extends to a census of all multiplicity-free fusion categories up to rank 7 under the assumption that the $\text {PSU}(2)_{12}$ fusion ring only has pivotal categorifications. The *F*-and *R*-symbols for the pivotal categories with $\text {PSU}(2)_{12}$ fusion rules were also obtained using alatc.

In the following, we will assume that the obtained census is an actual classification. Under this assumption, we can make the following general observations about the found fusion systems

**Fig. 3:**
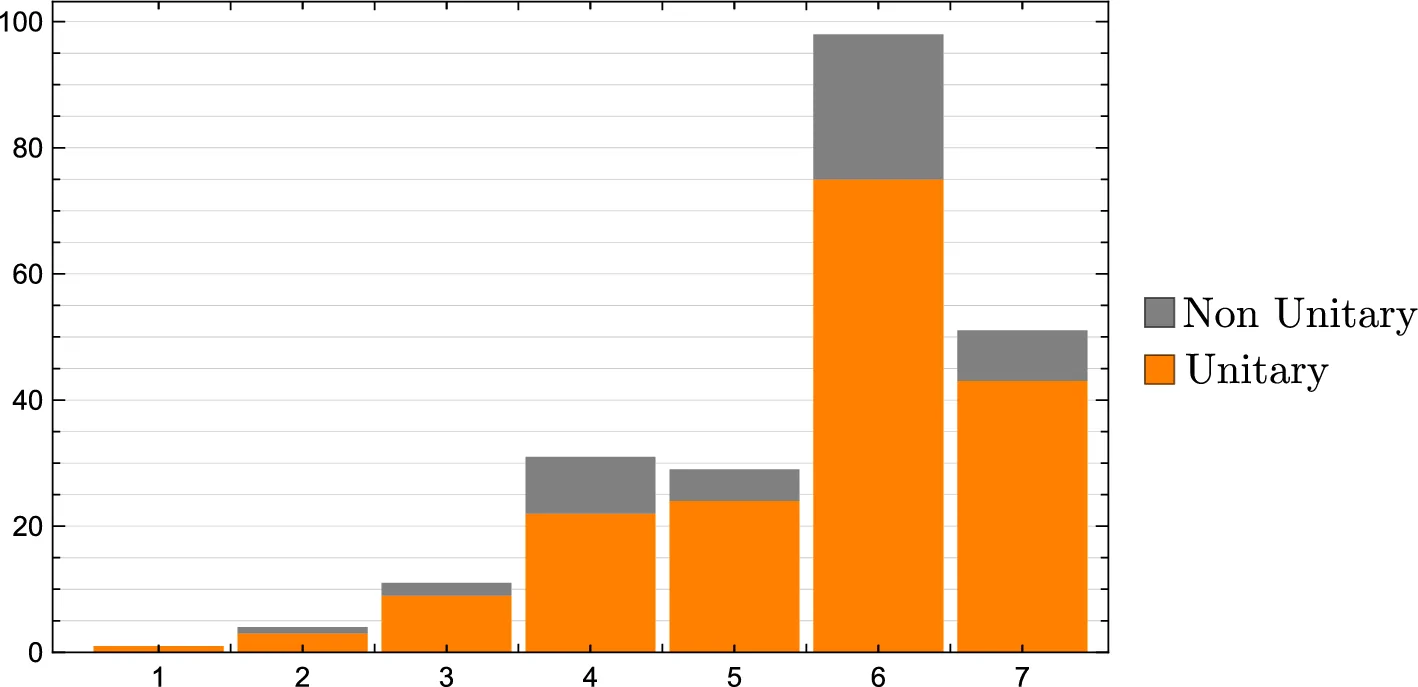
Number of solutions to the pentagon equations for multiplicity-free fusion rings per rank (horizontal axis). Unitary means that a gauge exists in which the *F*-symbols are unitary

**Fig. 4:**
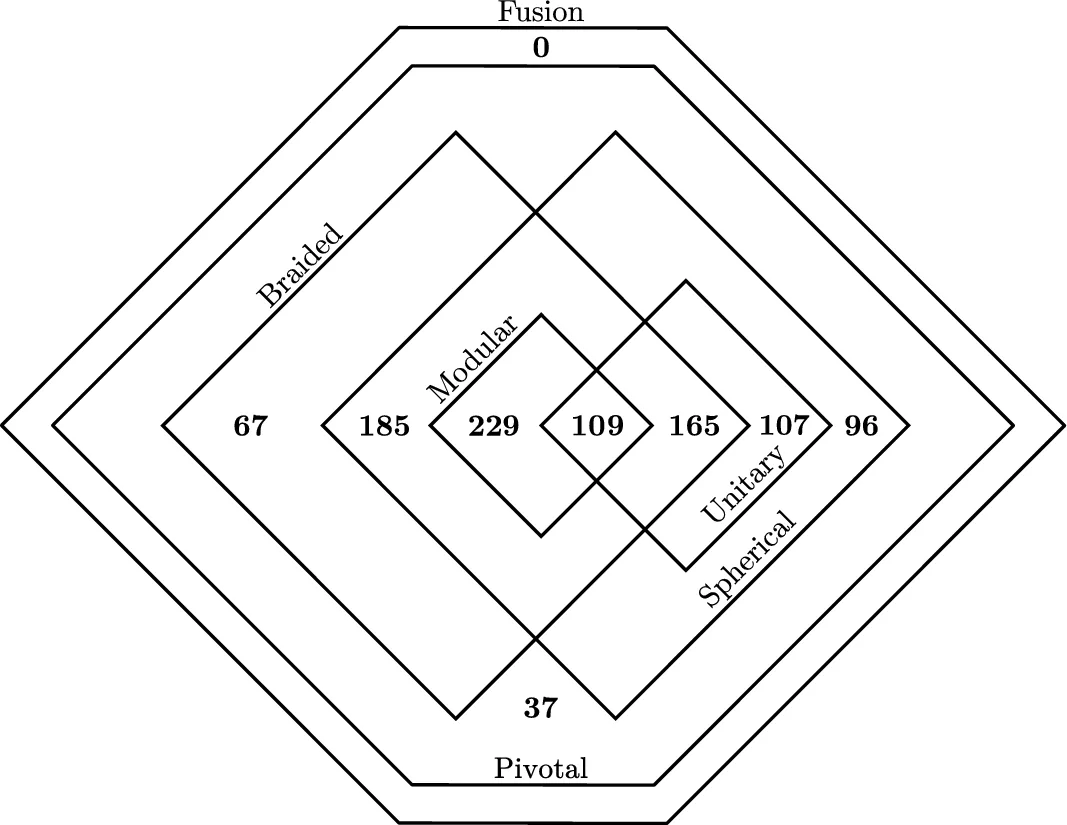
Venn diagram of the number of multiplicity-free fusion systems up to rank 7 with certain properties. By definition, the ribbon fusion systems belong to the intersection of the braided and spherical squares. Each number indicates the number of fusion systems that lie in the connected region of the Venn diagram. There are thus, e.g., 0 fusion systems with no compatible extra structures, 37 pivotal fusion systems with no compatible extra structures, 185 ribbon fusion systems that are neither modular nor unitary, 109 fusion systems that are modular and unitary, 229 fusion systems that are modular but non-unitary and thus 338 modular fusion systems in total

**Fig. 5:**
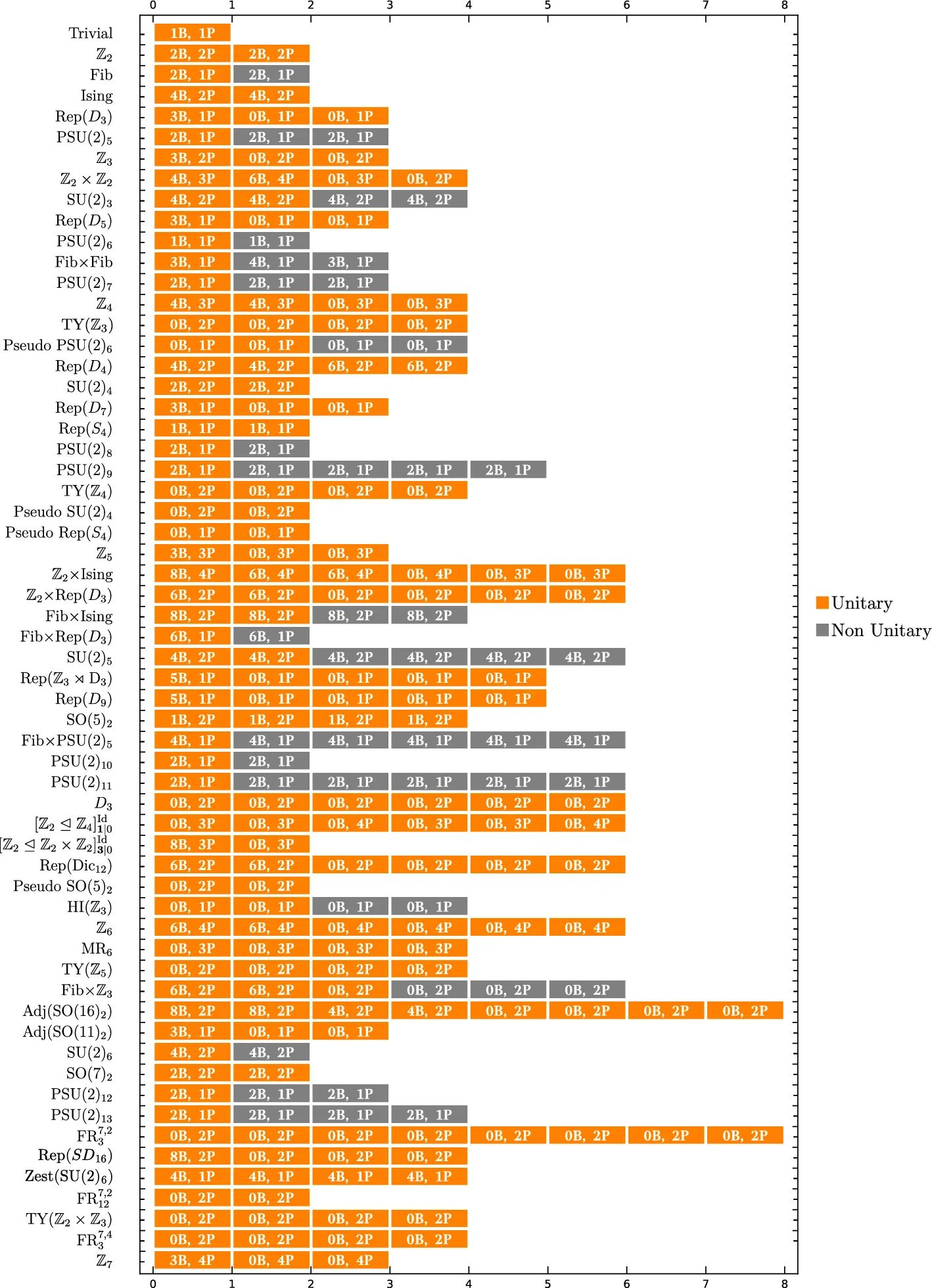
Number of solutions to the pentagon equations per fusion ring. Each rectangle corresponds to a unique fusion system. The first number in a rectangle denotes the number of braided fusion systems corresponding to that fusion system while the second denotes the number of pivotal fusion systems. Unitary (orange color) means a gauge exists in which the *F*-symbols are unitary, and **not** that all braided and pivotal fusion systems are unitary

- Out of the 115 multiplicity-free fusion rings of rank 7 or less, 60 are categorifiable, leading to a total of 225 inequivalent fusion systems (where only the data of the *F*-symbols is taken into account). All of these fusion systems have solutions to the pivotal equations, and 112 of the fusion systems admit a braided structure. In total, there are 989 inequivalent pivotal and possibly braided fusion systems. The number of inequivalent solutions to the pentagon equations per rank can be seen in Fig. 3. Figure 5 gives a complete overview of the number of pivotal and braided fusion systems per fusion ring, and Fig. 4 shows a Venn diagram of the number of systems that have a combination of any of the five properties: braided, unitary, spherical, ribbon, and modular. On the page on small multiplicity-free fusion categories on the anyonwiki, one can find a list of all multiplicity-free fusion categories with indicators for the existence of braided, unitary, spherical, ribbon, and modular structures.
- For all but three multiplicity-free fusion rings with rank up to seven there is a unique configuration of non-trivial zero values for the *F*-symbols. The three exceptions are the following.
  - $\text {FR}^{7,1,0}_1 = \text {Adj}(\text {SO}(16)_2)$ admits 56 (braided) pivotal fusion categories of which 32 have 192 *F*-symbols equal to 0, 16 have 96 *F*-symbols equal to 0, and 8 have 24 *F*-symbols equal to 0.
  - $\text {FR}^{7,1,0}_6 = \text {Adj}(\text {SO}(11)_2)$ has 5 categorifications of which 3 have 135 *F*-symbols equal to 0 and 2 have 45 *F*-symbols equal to 0.
  - $\text {FR}^{7,1,2}_4$ has 22 categorifications of which 16 have 192 *F*-symbols equal to 0, and 6 have 24 *F*-symbols equal to 0.
  For all three cases, the smaller sets of *F*-symbols that are zero are included in all larger sets of *F*-symbols that are zero, i.e., they form a chain of inclusions.
- Any categorifiable multiplicity-free fusion ring up to rank 7 has at least one fusion category that is either braided or unitary. Of course, as can be seen from Fig. 4, there are fusion categories that are neither unitary, nor braided.
- Any multiplicity-free fusion ring up to rank 7 for which modular data is available also categorifies into at least one modular category.
- The number of (pivotal) (braided) fusion systems of the product of two fusion rings is not necessarily equal to the product of the number of (pivotal) (braided) fusion systems for each ring separately. This can be seen from Fig. 5. For, e.g., the $\mathbb {Z}_2 \times \text {Ising}$ fusion ring one finds 6 solutions to the pentagon equations while the $\mathbb {Z}_2$ and $\text {Ising}$ fusion rings have 2 solutions each. Moreover, the number of braided fusion systems and the number of pivotal fusion systems for $\mathbb {Z}_2 \times \text {Ising}$ varies per solution to the pentagon equations, which is not the case for the individual fusion rings. There are even fusion systems for $\mathbb {Z}_2 \times \text {Ising}$ that cannot be braided at all, even though all fusion systems are braided for $\mathbb {Z}_2$, and $\text {Ising}$ separately.

Furthermore, almost all categorifiable fusion rings up to rank 7 belong to one of the following classes (note that these might overlap)

- Finite group rings:
  - $$\mathbb {Z}_k$$ for k=1,…,7,
  - $$\mathbb {Z}_2 \times \mathbb {Z}_2$$,
  - $$\mathbb {Z}_2 \times \mathbb {Z}_3$$,
  - $$D_3$$.
- Representation rings of finite groups:
  - $$\text {Rep}(D_{k})$$ for k=3,4,5,7,9,
  - $$\text {Rep}(S_4)$$,
  - $$\text {Rep}(\mathbb {Z}_3 \rtimes D_3)$$,
  - $$\text {Rep}(\text {Dic}_{12})$$,
  - $$\text {Rep}(SD_16)$$.
- Fusion rings coming from the representation theory of quantum groups at roots of unity:
  - $\text {PSU}(2)_k$ for k=3,…,13 (note that $\text {Fib} =\text {PSU}(2)_3$),
  - $\text {SU}(2)_k$ for k=2,…,6 (note that $\text {Ising} = \text {SU}(2)_2$),
  - $$\text {SO}(2k+1)_2$$ for k=1,2,3.
- Products of rings in one of the groups above:
  - $\mathbb {Z}_2\times \mathcal {R}$ with $\mathcal {R}$ any of the rings from the groups above with rank up to 3,
  - $\text {Fib}\times \mathcal {R}$ with $\mathcal {R}$ any of the rings from the groups above with rank up to 3 (note that $\text {TriCritIsing} = \text {Fib}\times \text {Ising}$).
- Extensions of rings in one of the groups above:
  - $\text {TY}(G)$ with *G* any commutative group ring up to rank 6,
  - $$\text {HI}(\mathbb {Z}_k)$$ for k=1,…,3.
- Zestings (see [36] for a definition) of rings in one of the groups above:
  - According to [25] , $\text {Pseudo SU}(2)_4 = \text {Zest}(\text {SU}(2)_4)$, $\text {Pseudo SO}(5)_2 = \text {Zest}(\text {SO}(5)_2)$, the three rings $[\mathbb {Z}_2 \trianglelefteq \mathbb {Z}_4]^{\text {Id}}_{\textbf{1}|0}$, $[\mathbb {Z}_2 \trianglelefteq \mathbb {Z}_2 \times \mathbb {Z}_2]^{\text {Id}}_{\textbf{3}|0}$, and $\text {MR}_6$ are zestings of $\mathbb {Z}_2 \times \text {Ising}$.
- Adjoint fusion rings (see [1] Chapter 3) of fusion rings coming from quantum groups at roots of unity: $\text {Adj}(\text {SO}(16)_2)$, $\text {Adj}(\text {SO}(11)_2)$.

The remaining fusion rings are the following: $\text {Pseudo PSU}(2)_6$, $\text {Pseudo Rep}(S_4)$, $\text {FR}^{7,2}_3$, $\text {FR}^{7,2}_{12}$, and $\text {FR}^{7,4}_3$.

According to [25], $\text {Pseudo PSU}(2)_6$ comes from the even part of the 1-supertransitive subfactor of index $3 + 2\sqrt{2}$ with non-self-adjoint objects [83].

The structure of the multiplication tables and the Frobenius-Perron dimensions (and private conversations with E. Rowell) indicate that $\text {FR}^{7,2}_{12}$ might be a zesting of $\text {SO}(7)_2$, and $\text {FR}^{7,2}_4$ might be a zesting of $\text {Adj}(\text {SO}(11)_2)$. From a conversation with S. Palcoux on Stackoverflow, we know that according to the paper [84] $\text {Pseudo Rep}(S_4)$, $\text {FR}^{7,2}_3$, and $\text {FR}^{7,4}_3$ should be *weakly group-theoretical*.

The list of fusion categories we found has been posted on the anyonwiki[26] website. There, one can also download all the numeric *F*-symbols, *R*-symbols and pivotal coefficients for each category. The symbolic data can be accessed as part of the Anyonica package, which is freely available.

## Data Availability

Snapshots of both the anyonwiki and Anyonica are available via their respective DOIs. The log files obtained as a result of the categorification are available upon request, as they occupy too much space for online storage.

## Other Software to Work with Fusion Rings and Fusion Categories

### Predecessors

Anyonica is partly based on software that has not been published. Bonderson’s thesis [85] describes in some detail basic techniques for solving multiplicity-free pentagon and hexagon equations on a computer, and we are using several of those. Slingerland wrote Mathematica code based on those techniques, and by using their code, they produced the tables of pentagon/hexagon solutions in the back of Bonderson’s thesis.

Subsequently, Slingerland found all multiplicity-free fusion rings up to rank 6 with a search algorithm similar to that used by Gepner [86] (but not specialized to rings with modular properties). He also found all solutions to the pentagon and hexagon equations for all categorifiable rings up to rank 6, although it was not clear that these were all solutions because unitarity was assumed in finding the zero *F*-symbols. Non-unitary systems with those same zeros patterns were also found, but there could have been systems with another configuration of zero *F*-symbols. Pivotal and spherical structures were not systematically considered. Neither the code nor the full results were published.

Verstraete also programmed software in Matlab to solve the pentagon and hexagon equations. Instead of fixing the gauge separately, a Smith decomposition was used to solve the logarithm of the binomial equations and to find a subspace of the solution set that is orthogonal to the space of gauge transforms. Under the assumption that a zero value can only appear for one *F*-symbol, the pentagon equations could also be solved numerically. To find a unitary gauge, the solver searched for a solution to the algebraic equations describing the unitarity of *F*-matrices. As far as we know, neither the code nor the results were published.

While Anyonica is written from scratch, it is still based on several techniques of Bonderson, Slingerland, and Verstraete. The method used for gauge fixing is the same as that of Slingerland and Bonderson. The reduction of systems of linear equations is based on that of Bonderson and Slingerland but has been generalized in the sense that Anyonica also divides by non-monomials. This requires more bookkeeping since assumptions need to be saved and checked recursively, but there is a payoff in the level of reduction that can be achieved. Anyonica’s method for solving binomial equations is the same as that of Verstraete, except that Anyonica fixes the gauge before solving these equations. While doing both in one go is more elegant, it is more efficient not to do so. Solving the eventual reduced system of equations is done the same way by every author, namely by using a built-in solver from Mathematica or Matlab based on Gröbner basis calculations.

Some of the aspects in which we think Anyonica improves upon its predecessors are

- The fact that no assumptions (in particular no unitarity) on the solutions are made,
- All configurations of 0 *F*-symbols can be found,
- Finding a unitary gauge, which used to be very hard, is now almost trivial,
- Any operation can be logged so it is easy to see when and why solutions are rejected,
- The code consists of modular pieces that can be used for any system of polynomial equations,
- The programming style is almost completely functional, so running the same code twice should result in the same outcome, and
- All fusion rings and categories (including pivotal, spherical, and modular data) are available, easy to access, easy to work with due to various extra functions, and are all combined into a single package.

There is still lots of functionality left to implement such an algorithm to compute the center of a category, check whether two categories are Morita-equivalent, find possible equivariantizations and de-equivartiantizations of a category, compute zestings of fusion categories, etc.

### Other Packages

Anyonica is not the only software that provides tools to work with fusion rings and fusion categories. Some of the other software packages are the following

- Mäurer and Thiel are developing TensorCategories.jl [79, 87, 88], a package that provides a framework as well as examples for computations in the realm of categories. Recently an algorithm has been added to compute the center of a fusion category. With help from the first author, the data of Anyonica has been imported to TensorCategories.jl and has been used by Mäurer and Thiel to find the centers of all multiplicity-free fusion categories up to rank 5.
- Kac is a software package that computes fusion rules for rational conformal field theories based on affine Lie algebras. It can be accessed at https://www.nikhef.nl/~t58/Site/Kac.html.
- Recently, Aboumrad released a software package that solves the pentagon and hexagon equations for specific fusion rings [89]. The functionality is more oriented towards applications in topological quantum computation and, at the moment, seems only to categorify multiplicity-free fusion rings coming from the representation theory of quantum groups at roots of unity. The nice thing about the software is that it is entirely open-source, uses parallelization, and can be paused and resumed in a user-friendly way at any time. It also cleverly handles the creation of the pentagon equations: these are constructed at the same time they are being solved. At the moment, Anyonica creates all the equations in one go, and then it starts simplifying. This leads to unnecessary memory consumption, so we hope to implement a more clever method soon. The way that pentagon and hexagon equations are being dealt with is clever, but some assumptions, such as the existence of braiding and orthogonality of the *F*-symbols, do not hold for general fusion categories.
- There is another package for finding fusion categories related to the representation theory of quantum groups at roots of unity: alatc (affine Lie algebra and tensor categories), written by Ardonne [90, 91]. This package finds fusion categories and braided structures based on calculating Clebsch-Gordan coefficients. It can handle fusion rings with multiplicity but comes with the downside that, just like Anyonica, it is implemented in the Wolfram Language. It can find *F*-symbols for fusion rings that are much larger than Anyonica is capable of handling. There are plans to incorporate alatc into Anyonica.
- Hagge and Titsworth constructed a Mathematica package (referenced in the introduction of [77]) that contains data on various categories, including some of rank 8 and higher. It also contains data for rigidity, pivotal, and braiding structures, calculates *S*-and *T*-matrices, and can check for monoidal equivalence of two categories.
- Bridgeman created the GitHub repository “smallRankUnitaryFusionData” [92], which contains data on all multiplicity-free unitary fusion categories (and their braidings) up to rank six. This data has also been incorporated in the TensorKit.jl [93] package from Haegeman via the extension CategoryData.jl [94] from Devos.
- The SageMath code to check various categorifiablity criteria listed in 4.1.1 can be found in the paper [25].
- For the classification of modular data of integral fusion rings up to rank 13 [60] the Normaliz software was used. The integrality constraint puts bounds on the sums of certain fusion coefficients, and Normaliz is very fast at finding all integer lattice points within a polytope defined by such bounds.

## Footnotes

1 Different authors might have different opinions on what should be regarded as *standard*.

2 There are several subtly different notions of dimension of a fusion category. Rather than defining all of those, we refer to the papers themselves for the appropriate definitions.

3 This might require exporting symbolic expressions in Mathematica to equivalent ones in another language, however.
